# Microbial biogeography of 925 geothermal springs in New Zealand

**DOI:** 10.1038/s41467-018-05020-y

**Published:** 2018-07-23

**Authors:** Jean F. Power, Carlo R. Carere, Charles K. Lee, Georgia L. J. Wakerley, David W. Evans, Mathew Button, Duncan White, Melissa D. Climo, Annika M. Hinze, Xochitl C. Morgan, Ian R. McDonald, S. Craig Cary, Matthew B. Stott

**Affiliations:** 1 Geomicrobiology Research Group, Department of Geothermal Sciences, GNS Science, Taupō, 3384 New Zealand; 2 Thermophile Research Unit, School of Science, University of Waikato, Hamilton, 3240 New Zealand; 3 Department of Chemical and Process Engineering, University of Canterbury, Christchurch, 8140 New Zealand; 4 Department of Computer Science, University of Waikato, Hamilton, 3240 New Zealand; 5 Wairakei Research Centre, GNS Science, Taupō, 3384 New Zealand; 6 School of Biological Sciences, University of Canterbury, Christchurch, 8140 New Zealand; 7 Department of Microbiology and Immunology, University of Otago, Dunedin, 9054 New Zealand

## Abstract

Geothermal springs are model ecosystems to investigate microbial biogeography as they represent discrete, relatively homogenous habitats, are distributed across multiple geographical scales, span broad geochemical gradients, and have reduced metazoan interactions. Here, we report the largest known consolidated study of geothermal ecosystems to determine factors that influence biogeographical patterns. We measured bacterial and archaeal community composition, 46 physicochemical parameters, and metadata from 925 geothermal springs across New Zealand (13.9–100.6 °C and pH < 1–9.7). We determined that diversity is primarily influenced by pH at temperatures <70 °C; with temperature only having a significant effect for values >70 °C. Further, community dissimilarity increases with geographic distance, with niche selection driving assembly at a localised scale. Surprisingly, two genera (*Venenivibrio* and *Acidithiobacillus*) dominated in both average relative abundance (11.2% and 11.1%, respectively) and prevalence (74.2% and 62.9%, respectively). These findings provide an unprecedented insight into ecological behaviour in geothermal springs, and a foundation to improve the characterisation of microbial biogeographical processes.

## Abstract (web summary)

Power et al. catalogue the microbial biodiversity and physicochemistry of around 1000 hotsprings across New Zealand, providing insights into the ecological conditions that drive community assembly in these ecosystems.

## Introduction

Biogeography identifies patterns of diversity across defined spatial or temporal scales in an attempt to describe the factors which influence these distributions. Recent studies have shown that microbial community diversity is shaped across time and space^1,2^ via a combination of environmental selection, stochastic drift, diversification, and dispersal limitation^3,4^. The relative impact of these ecological drivers on diversity is the subject of ongoing debate, with differential findings reported across terrestrial, marine, and human ecosystems^5–8^.

Geothermally-heated springs are ideal systems to investigate microbial biogeography, because, in comparison to terrestrial environments, they represent discrete, aquatic habitats with broad geochemical and physical gradients distributed across proximal and distal geographic distances. The relatively constrained microbial community structures, typical of geothermal springs compared to soils and sediments, also allow for the robust identification of diversity trends. Separate studies have each implicated temperature^9–11^, pH^12^, and seasonality^13^ as the primary drivers of bacterial and archaeal communities in these ecosystems; with niche specialisation observed within both local and regional populations^14,15^. Other geochemical variables, particularly hydrogen^16,17^ and nitrogen^18,19^, may also contribute to community structure. Concurrently, the stochastic action of microbial dispersal is thought to be a significant driver behind the distribution of microorganisms^20^, with endemism and allopatric speciation reported in intercontinental geothermal springs^21,22^. It is important to note that significant differences have been found between aqueous and soil/sediment samples from the same springs^10,12,23^; emphasising that the increased relative homogeneity of aqueous samples make geothermal water columns excellent candidate environments for investigating large-scale taxa–geochemical associations. Despite these findings, a lack of sampling quantity/density and physicochemical gradient scales, uniformity in sampling methodology, and a concurrent assessment of geographic distance, within a single study, has hindered a holistic view of microbial biogeography from developing.

**Fig. 1:**
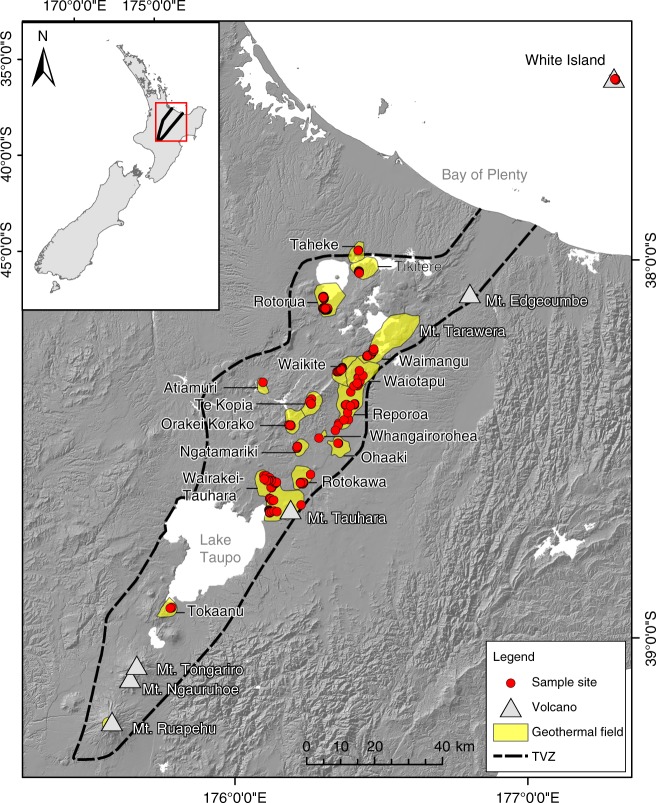
Map of the Taupō Volcanic Zone (TVZ), New Zealand. The geothermal fields from which samples were collected are presented in yellow. All sampled geothermal springs (*n* = 1019) are marked by red circles. The panel insert highlights the location of the TVZ in the central north island of New Zealand. The topographic layers for this map were obtained from Land Information New Zealand (LINZ; CC-BY-4.0) and the TVZ boundary defined using data from Wilson et al.^24^

The Taupō Volcanic Zone (TVZ) is a region rich in geothermal springs and broad physicochemical gradients spanning 8000 km^2^ in New Zealand’s North Island (Fig. 1), making it a tractable model system for studying microbial biogeography. The area is a rifting arc associated with subduction at the Pacific-Australian tectonic plate boundary, resulting in a locus of intense magmatism^24^. The variable combination of thick, permeable volcanic deposits, high heat flux, and an active extensional (crustal thinning) setting favours the deep convection of groundwater and exsolved magmatic volatiles that are expressed as physicochemically-heterogeneous surface features in geographically distinct geothermal fields^25^. Previous microbiological studies across the region have hinted at novel diversity and function present within some of these features^26–30^, however investigations into the biogeographical drivers within the TVZ are sparse and have focused predominantly on soil/sediments or individual hotsprings^9,11,31^.

Here, we report the diversity and biogeography of microbial communities found in geothermal springs, collected as part of the 1000 Springs Project. This project aimed to catalogue the microbial biodiversity and physicochemistry of New Zealand geothermal springs to serve as a conservation, scientific, and indigenous cultural knowledge repository; a publicly accessible database of all springs surveyed is available online (http://1000Springs.org.nz). In order to determine the influence of biogeographical processes on bacterial and archaeal diversity and community structure within geothermal springs, we collected water-columns and metadata from 1019 spring samples within the TVZ over a 21-month period using rigorously standardised methodologies. We then performed community analysis of the bacterial and archaeal population (16S rRNA gene amplicon sequencing) and quantified 46 physicochemical parameters for each sample. This work represents the largest known microbial ecology study on geothermal aquatic habitats at a regional scale and complements a parallel study on protist diversity in New Zealand geothermal springs^32^. Our results demonstrate both the relative influence of physicochemical parameters (e.g. pH) and the effect of geographic isolation on the assemblage of communities in these extreme ecosystems. Collectively, these findings expand our knowledge of the constraints that govern universal microbial biogeographical processes.

## Results and Discussion

### Geothermal feature sampling

Recent biogeography research has demonstrated that microbial diversity patterns are detectable and are influenced by both deterministic^6^ and stochastic processes^7^. A lack of consensus on the relative impact of these factors, however, has been exacerbated by the absence of data across broad physicochemical gradients, and sampling scales and density across both geographic distance and habitat type. The inherent heterogeneity of terrestrial soil microbial ecosystems^33^ further confounds attempts to distinguish true taxa–geochemical associations. To provide greater resolution to the factors driving microbial biogeography processes, we collected 1019 geothermal water-column samples from across the TVZ and assessed physicochemical and microbial community composition (Fig. 1). Samples included representatives of both extreme pH (< 0–9.7) and temperature (13.9–100.6 °C) (Supplementary Fig. 1). The filtering of low-quality and temporal samples yielded a final data set of 925 individual geothermal springs for spatial-statistical analysis (details can be found in Supplementary Methods). From these 925 springs, a total of 28,381 operational taxonomic units (OTUs; 97% similarity) were generated for diversity studies.

### Microbial diversity is principally driven by pH, not temperature

Reduced microbial diversity in geothermal springs is often attributed to the extreme environmental conditions common to these areas. Temperature and pH are reported to be the predominant drivers of microbial diversity^9,34^, but their influence relative to other parameters has not been investigated over large geographic and physicochemical scales with appropriate sample density. Our analysis of microbial richness and diversity showed significant variation spanning pH, temperature, and geographical gradients within the TVZ (richness: 49–2997 OTUs, diversity: 1.1–7.3 Shannon index; Supplementary Figs. 2 and 3). As anticipated, average OTU richness (386 OTUs; Supplementary Fig. 4) was reduced in comparison to studies of non-geothermal temperate terrestrial^35^ and aquatic^1^ environments. Further, OTU richness was maximal at the geothermally-moderate temperature of 21.5 °C and at circumneutral pH 6.4. This is consistent with the hypothesis that polyextreme habitats prohibit the growth of most microbial taxa, a trend reported in both geothermal and non-geothermal environments alike^5,9^. A comparison of 46 individual physicochemical parameters (Supplementary Table 1) confirmed pH as the most significant factor influencing diversity (16.4%, linear regression: *P* < 0.001; Supplementary Fig. 3), with diversity increasing from acidic to alkaline pH. Further, multiple regression analysis showed ${\mathrm{NO}}_3^ -$, turbidity (TURB), oxidation–reduction potential (ORP), dissolved oxygen (dO), ${\mathrm{NO}}_2^ -$, Si, and Cd also had meaningful contributions (Supplementary Table 2). Cumulatively, along with pH, these factors accounted for 26.6% of the observed variation in Shannon diversity. Correlation of pH with Shannon index (Pearson’s coefficient: |*r*| = 0.41, *P* < 0.001) and significance testing between samples binned by pH increments (Kruskal–Wallis: *H* = 179.4, *P* < 0.001; Supplementary Fig. 1) further confirmed pH as a major driver of variation in alpha diversity. This finding is consistent with reports of pH as the primary environmental predictor of microbial diversity in several ecosystems, both in New Zealand and globally (e.g. soil^5,36^, water^32,37^, alpine^38,39^).

**Fig. 2:**
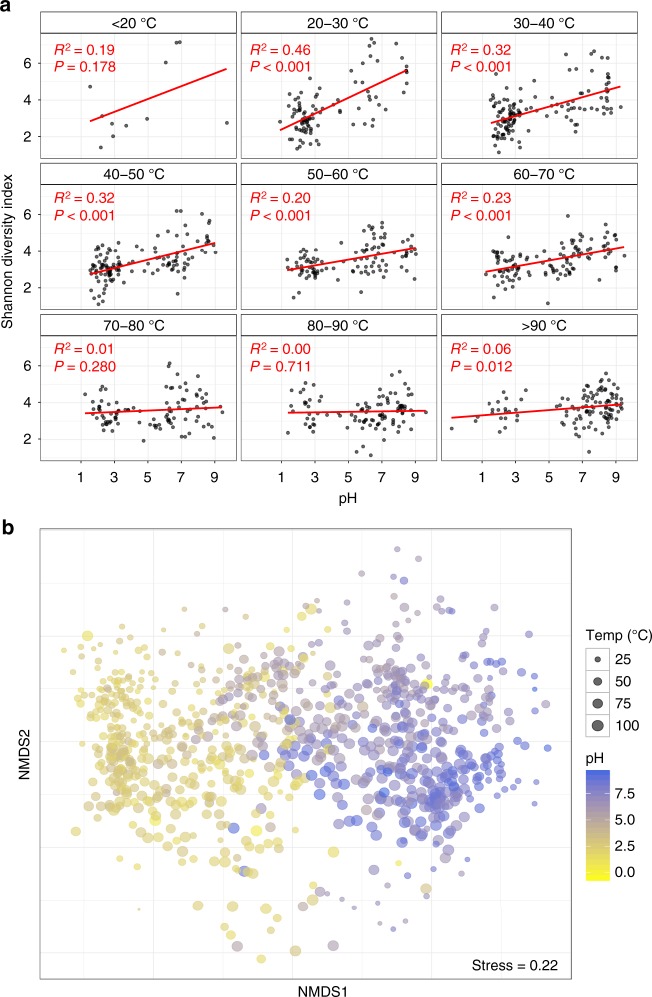
Alpha and beta diversity as a function of pH and temperature. **a** pH against alpha diversity via Shannon index of all individual springs (*n* = 925) in 10 °C increments, with linear regression applied to each increment. **b** Non-metric multidimensional scaling (NMDS) plot of beta diversity (via Bray–Curtis dissimilarities) between all individual microbial community structures sampled (*n* = 925)

It has been previously hypothesised that pH has significant influence on microbial community composition because changes in proton gradients will drastically alter nutrient availability, metal solubility, or organic carbon characteristics^5^. Similarly, acidic pH will also reduce the number of taxa observed due to the decreased number that can physiologically tolerate these conditions^40^ compared to non-acidic habitats. Here, we demonstrate that pH had the most significant effect on diversity across all springs measured, but due to our high sampling frequency, we see this influence diminishes at temperatures > 70 °C (Fig. 2). Inversely, the effect of temperature on diversity was lessened in springs where pH was < 4 (Supplementary Fig. 5). There is some evidence that suggests thermophily predates acid tolerance^40,41^, thus it is possible the added stress of an extreme proton gradient across cell membranes has constrained the diversification of the thermophilic chemolithoautotrophic organisms common to these areas^42^. Indeed, a recent investigation of thermoacidophily in Archaea suggests hyperacidophily (growth < pH 3.0) may have only arisen as little as ~0.8 Ga^40^, thereby limiting the opportunity for microbial diversification; an observation highlighted by the paucity of these microorganisms in extremely acidic geothermal ecosystems^11,40^. It is also important to note that salinity has previously been suggested as an important driver of microbial community diversity^43,44^. The quantitative data in this study showed only minimal influence of salinity (proxy as conductivity (COND)) on diversity (linear regression: *R*^2^ = 0.001, *P* = 0.2720; Supplementary Table 1), bearing in mind that the majority of the geothermal spring samples in this study had salinities substantially less than that of seawater.

The relationship between temperature and alpha diversity reported in this research starkly contrasts a previous intercontinental study comparing microbial community diversity in soil/sediments from 165 geothermal springs^9^, which showed that a strong relationship (*R*^2^ = 0.40–0.44) existed. In contrast, our data across the entire suite of samples, revealed that temperature had no significant influence on observed community diversity (*R*^2^ = 0.002, *P* = 0.201; Supplementary Fig. 3, Supplementary Table 1). This result increased marginally for archaeal-only diversity (*R*^2^ = 0.013, *P* = 0.0005), suggesting that temperature has a more profound effect on this domain than it does on bacteria. However, the primers used in this study are known to be unfavourable towards some archaeal clades^45^, therefore it is likely that extensive archaeal diversity remains undetected in this study. The lack of influence of temperature on whole community diversity was further substantiated via multiple linear modelling (Supplementary Table 2), and significance and correlation testing (Kruskal–Wallis: *H* = 16.2, *P* = 0.039; Pearson’s coefficient: |*r*| = 0.04, *P* = 0.201). When samples were split into pH increments, like Sharp et al.^9^, we observed increasing temperature only significantly constrained diversity above moderately acidic conditions (pH > 4; Supplementary Fig. 5). However, the magnitude of this effect was, in general, far less than previously reported and is likely a consequence of the sample type (e.g. soil/sediments versus aqueous) and density of samples processed^12^. Many samples from geothermal environments are recalcitrant to traditional DNA extraction protocols and research in these areas has therefore focused on those with greater biomass abundance^9,34^ (i.e. soils, sediments, streamers, or biomats). Whereas aqueous samples typically exhibit a more homogenous chemistry and community structure, the heterogeneity of terrestrial samples is known to affect microbial population (e.g. particle size, depth, nutrient composition)^33^. Our deliberate use of aqueous samples extends the results of previous small-scale work^10,31^ and also permits the robust identification of genuine taxa–geochemical relationships in these environments.

### Microbial communities are influenced by pH, temperature, and source fluid

**Fig. 3:**
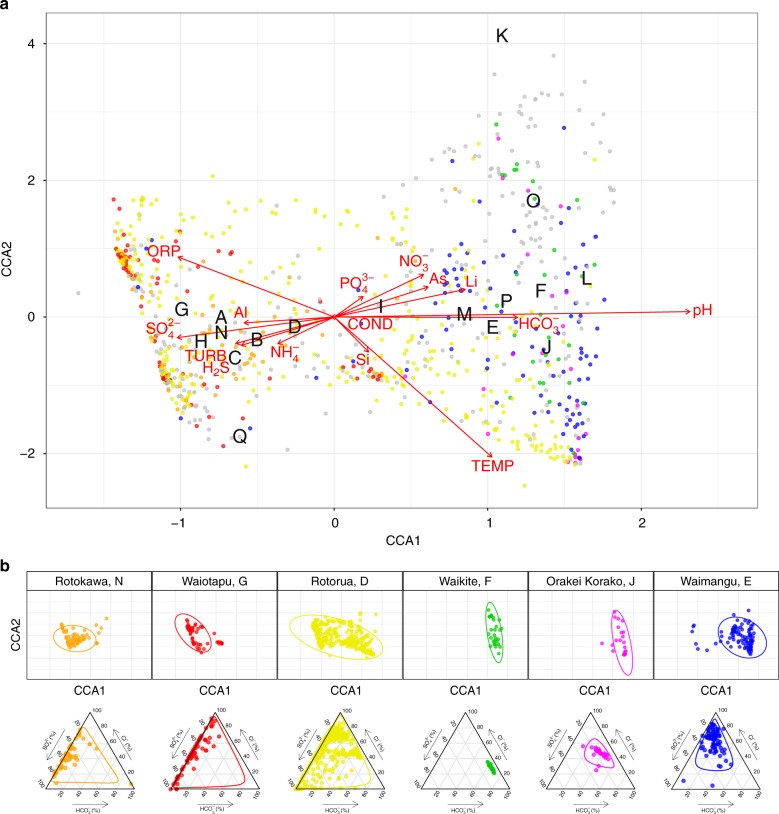
Constrained correspondence analysis (CCA) of beta diversity with significant physicochemistry. **a** A scatter plot of spring community dissimilarities (*n* = 923), with letters corresponding to centroids from the model for geothermal fields (A–Q; White Island, Taheke, Tikitere, Rotorua, Waimangu, Waikite, Waiotapu, Te Kopia, Reporoa, Orakei Korako, Whangairorohea, Ohaaki, Ngatamariki, Rotokawa, Wairakei-Tauhara, Tokaanu, Misc). Coloured communities are from fields represented in the subpanel. Constraining variables are plotted as arrows (COND: conductivity, TURB: turbidity), with length and direction indicating scale and area of influence each variable had on the model. **b** The top panel represents a subset of the full CCA model, with select geothermal fields shown in colour (including 95% confidence intervals). The bottom panel shows their respective geochemical signatures as a ratio of chloride (Cl^−^), sulfate $\left( {{\mathrm{SO}}_4^{2 - }} \right)$, and bicarbonate $\left( {{\mathrm{HCO}}_3^ - } \right)$

Throughout the TVZ, beta diversity correlated more strongly with pH (Mantel: *ρ* = 0.54, *P* < 0.001) than with temperature (Mantel: *ρ* = 0.19, *P* < 0.001; Fig. 2, Supplementary Table 3). This trend was consistent in pH- and temperature-binned samples (ANOSIM: |*R*| = 0.46 and 0.18, respectively, *P* < 0.001; Supplementary Fig. 6); further confirming pH, more so than temperature, accounted for observed variations in beta diversity. Congruent with our finding that pH influences alpha diversity at lower temperatures (< 70 °C), the effect of temperature reducing beta diversity had greater significance above 80 °C (Wilcox: *P* < 0.001; Supplementary Fig. 6). The extent of measured physicochemical properties across 925 individual habitats, however, allowed us to explore the environmental impact on community structures beyond just pH and temperature. Permutational multivariate analysis of variance in spring community assemblages showed that pH (12.4%) and temperature (3.9%) had the greatest contribution towards beta diversity, followed by ORP (1.4%), ${\mathrm{SO}}_4^{2 - }$ (0.8%), TURB (0.8%), and As (0.7%) (*P* < 0.001; Supplementary Table 4). Interestingly, constrained correspondence analysis of the 15 most significant, non-collinear, and variable parameters (Supplementary Tables 4 and 5; pH, temperature, TURB, ORP, ${\mathrm{SO}}_4^{2 - }$, ${\mathrm{NO}}_3^ -$, As, ${\mathrm{NH}}_4^ +$, ${\mathrm{HCO}}_3^ -$, H_2_S, COND, Li, Al, Si, and ${\mathrm{PO}}_4^{3 - }$), along with geothermal field locations, only explained 10% of variation in beta diversity (Fig. 3), indicating physicochemistry, or at least the 46 parameters measured, were not the sole drivers of community composition.

**Fig. 4:**
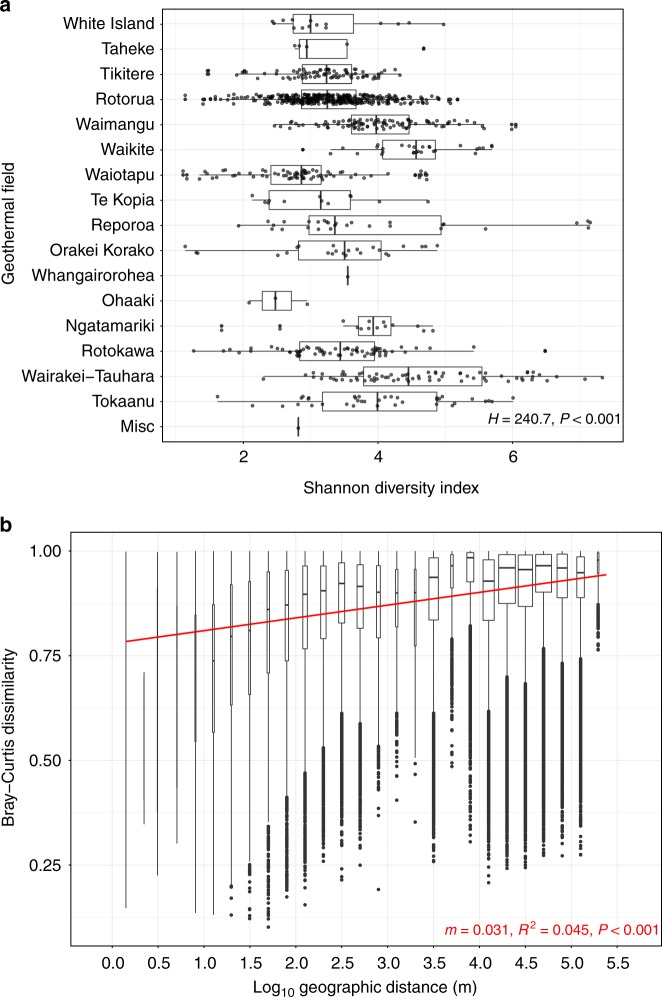
Alpha and beta diversity as a function of geographic distance. **a** Alpha diversity scales (via Shannon index) across individual springs (*n* = 925), split by geothermal fields. Fields are ordered from north to south (*H*: Kruskal–Wallis test). **b** A distance-decay pattern of beta diversity (via Bray–Curtis dissimilarities of 925 springs) against pairwise geographic distance in metres, with linear regression applied (*m* = slope). Geographic distance is split into bins to aid visualisation of the spread

Geothermal fields are known to express chemical signatures characteristic of their respective source fluids^46^, implying autocorrelation could occur between location and geochemistry. We therefore investigated whether typical geochemical conditions exist for springs within the same geothermal field and whether specific microbial community assemblages could be predicted. Springs are usually classified according to these source fluids; alkaline-chloride or acid-sulfate. High-chloride features are typically sourced from magmatic waters and have little interaction with groundwater aquifers. At depth, water–rock interactions can result in elevated bicarbonate concentrations and, consequently, neutral to alkaline pH in surface features. Acid-sulfate springs (< pH 3), in contrast, form as steam-heated groundwater couples with the eventual oxidation of hydrogen sulfide into sulfate (and protons). Rarely, a combination of the two processes can occur; leading to intermediate pH values^47^. It is unknown, however, whether these source fluid characteristics are predictive of their associated microbial ecosystems. Bray–Curtis dissimilarities confirmed that, like alpha diversity (Kruskal–Wallis: *H* = 240.7, *P* < 0.001; Fig. 4), community structures were significantly different between geothermal fields (ANOSIM: |*R*| = 0.26, *P* < 0.001; Supplementary Fig. 7). Gradient analysis comparing significant geochemical variables and geography further identified meaningful intra-geothermal field clustering of microbial communities (95% CI; Fig. 3 and Supplementary Fig. 8). Further, characteristic geochemical signatures from these fields were identified and analysis suggests they could be predictive of community composition. For example, the Rotokawa and Waikite geothermal fields (approx. 35 km apart) (Fig. 3 position N and F) display opposing ratios of ${\mathrm{HCO}}_3^ -$, ${\mathrm{SO}}_4^{2 - }$, and Cl^−^, with corresponding microbial communities for these sites clustering independently in ordination space. Despite this association, intra-field variation in both alpha and beta diversity also occurred at other geothermal sites where geochemical signatures were not uniform across local springs (e.g. Rotorua, Fig. 3 position D), demonstrating that correlation does not necessarily always occur between locational proximity and physicochemistry.

### Aquificae and Proteobacteria are abundant and widespread

**Fig. 5:**
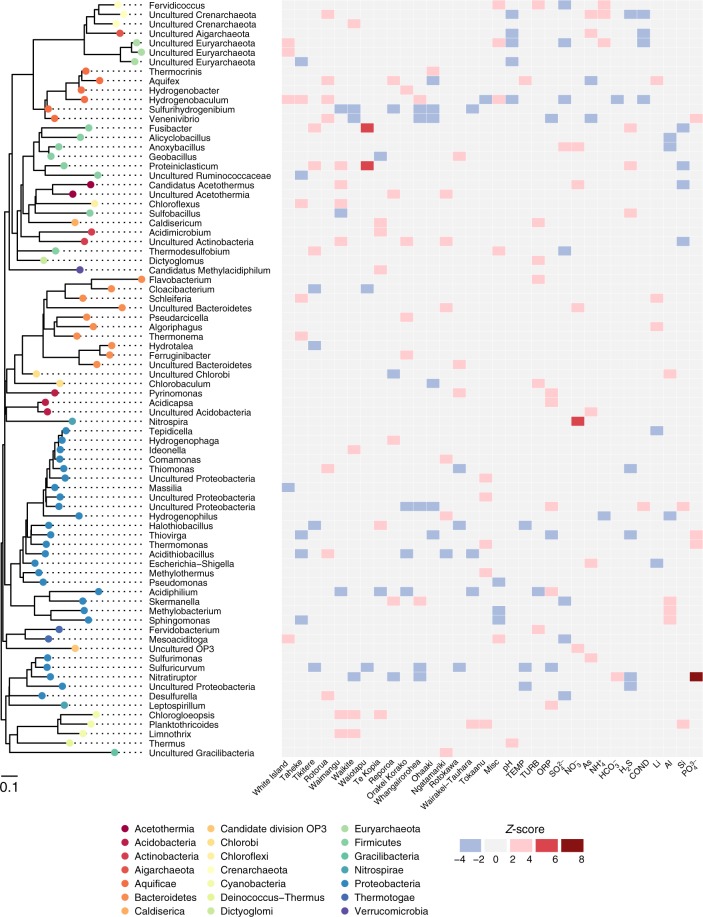
Taxonomic association with location and physicochemistry. The heat map displays positive (red) and negative (blue) association of genus-level taxa (> 0.1% average relative abundance) with each geothermal field and significant environmental variables, based on *Z*-scores of abundance log ratios. Each taxon is colour-coded to corresponding phylum on the approximately maximum-likelihood phylogenetic tree

To determine whether individual microbial taxa favoured particular environmental conditions and locations, we first assessed the distribution of genera across all individual springs. Within 17 geothermal fields and 925 geothermal features, 21 phyla were detected with an average relative abundance > 0.1% (Fig. 5). We found that two phyla and associated genera, Proteobacteria (*Acidithiobacillus* spp.) and Aquificae (*Venenivibrio, Hydrogenobaculum*, *Aquifex* spp.), dominated these ecosystems (65.2% total average relative abundance across all springs), composing nine of the 15 most abundant genera > 1% average relative abundance (Table 1). Considering the broad spectrum of geothermal environmental conditions sampled in this study (we assessed microbial communities in springs across a pH gradient of nine orders of magnitude and a temperature range of ~87 °C) and the prevalence of these taxa across the region, this result was surprising. Proteobacteria was the most abundant phylum across all samples (34.2% of total average relative abundance; Table 1), found predominantly at temperatures less than 50 °C (Supplementary Fig. 9). Of the 19 most abundant proteobacterial genera (average relative abundance > 0.1%), the majority are characterised as aerobic chemolithoautotrophs, utilising either sulfur species and/or hydrogen for metabolism. Accordingly, the most abundant (11.1%) and prevalent (62.9%) proteobacterial genus identified was *Acidithiobacillus*^48^, a mesophilic-moderately thermophilic, acidophilic autotroph that utilises reduced sulfur compounds, iron, and/or hydrogen as energy for growth.

**Table 1:** Average relative abundance and prevalence of phyla and genera

Phylum	Genus	Abundance	SD	Max	Prevalence
Aquificae	*Venenivibrio*	0.112	0.231	0.968	0.742
Proteobacteria	*Acidithiobacillus*	0.111	0.242	0.994	0.629
Aquificae	*Hydrogenobaculum*	0.100	0.235	0.999	0.608
Aquificae	*Aquifex*	0.086	0.212	0.971	0.497
Deinococcus-Thermus	*Thermus*	0.025	0.071	0.732	0.552
Proteobacteria	*Thiomonas*	0.024	0.101	0.941	0.396
Proteobacteria	*Desulfurella*	0.022	0.067	0.758	0.497
Crenarchaeota	Sulfolobaceae (f)	0.020	0.091	0.951	0.416
Euryarchaeota	Thermoplasmatales (o)	0.019	0.059	0.495	0.539
Proteobacteria	*Thiovirga*	0.015	0.077	0.816	0.374
Proteobacteria	Hydrogenophilaceae (f)	0.015	0.072	0.704	0.406
Thermodesulfobacteria	*Caldimicrobium*	0.015	0.052	0.651	0.519
Proteobacteria	*Hydrogenophilus*	0.013	0.045	0.432	0.484
Thermotogae	*Mesoaciditoga*	0.011	0.033	0.286	0.410
Parcubacteria	Parcubacteria (p)	0.010	0.024	0.193	0.608

Only taxa above a 1% average compositional threshold are shown. Maximum abundance of each taxon within individual features and standard deviation across all 925 springs. Where taxonomy assignment failed to classify to genus level, the closest assigned taxonomy is shown

SD = standard deviation, f = family, o = order, p = phylum

Aquificae (order Aquificales) was the second most abundant phylum overall (31% average relative abundance across 925 springs) and included three of the four most abundant genera; *Venenivibrio*, *Hydrogenobaculum*, and *Aquifex* (11.2%, 10.0%, and 8.6%, respectively; Table 1). As Aquificae are thermophilic (*T*_opt_ 65–85 °C)^49^, they were much more abundant in warmer springs (> 50 °C; Supplementary Fig. 9). The minimal growth temperature reported for characterised Aquificales species (*Sulfurihydrogenibium subterraneum* and *Sulfurihydrogenibium kristjanssonii*)^49^ is 40 °C and may explain the low Aquificae abundance found in springs less than 50 °C. Terrestrial Aquificae are predominately microaerophilic chemolithoautotrophs that oxidise hydrogen or reduced sulfur compounds; heterotrophy is also observed in a few representatives^49^. Of the 14 currently described genera within the Aquificae, six genera were relatively abundant in our dataset (average relative abundance > 0.1%; Fig. 5): *Aquifex*, *Hydrogenobacter*, *Hydrogenobaculum* and *Thermocrinis* (family Aquificaceae), and *Sulfurihydrogenibium* and *Venenivibrio* (family Hydrogenothermaceae). No signatures of the Desulfurobacteriaceae were detected and is consistent with reports that all current representatives from this family are associated with deep-sea or coastal thermal vents^49^. *Venenivibrio* (OTUs; *n* = 111) was also the most prevalent and abundant genus across all communities (Table 1). This taxon, found in 74.2% (*n* = 686) of individual springs sampled, has only one cultured representative, *Venenivibrio stagnispumantis* (CP.B2^T^), which was isolated from the Waiotapu geothermal field in the TVZ^29^. The broad distribution of this genus across such a large number of habitats was surprising, as growth of the type strain is only supported by a narrow set of conditions (pH 4.8–5.8, 45–75 °C). Considering this, and the number of *Venenivibrio* OTUs detected, we interpret this result as evidence there is substantial undiscovered phylogenetic and physiological diversity within the genus. The ubiquity of *Venenivibrio* suggests that either the metabolic capabilities of this genus extend substantially beyond those described for the type strain, and/or that many of the divergent taxa could be persisting and not growing under conditions detected in this study^30,50^.

### Geochemical and geographical associations exist at the genus level

The two most abundant phyla, Proteobacteria and Aquificae, were found to occupy a characteristic ecological niche (< 50 and > 50 °C, respectively, Supplementary Fig. 9). To investigate specific taxa–geochemical associations beyond just temperature and pH, we applied a multivariate linear model to determine enrichment of taxa in association with geothermal fields and other environmental data (Fig. 5). The strongest associations between taxa and chemistry (*Z*-score > 4) were between *Nitrospira*–nitrate $\left( {{\mathrm{NO}}_3^ - } \right)$ and *Nitratiruptor*–phosphate $\left( {{\mathrm{PO}}_4^{3 - }} \right)$. *Nitrospira* oxidises nitrite to nitrate and therefore differential high abundance of this taxon in nitrate-rich environments is expected. Further, the positive *Nitratiruptor*–${\mathrm{PO}}_4^{3 - }$ relationship suggests phosphate is a preferred nutritional requirement for this chemolithoautotroph^51^ and informs future efforts to isolate members of this genus would benefit from additional phosphate or the presence of reduced phosphorous compounds in the culture medium^52,53^. *Thermus* and *Hydrogenobaculum* were the only bacterial taxa to differentially associate (compared to other taxa) positively and negatively with pH respectively. This is consistent with the lack of acidophily phenotype (pH < 4) reported in *Thermus* spp.^54^ and conversely, the preferred acidic ecological niche of *Hydrogenobaculum*^55^. *Aquifex* was the only genus to display above average association with temperature, confirming abundance of this genera is significantly enhanced by hyperthermophily^56^.

Similar to the chemical–taxa associations discussed above, differential abundance relationships were calculated with respect to individual geothermal fields (Fig. 5). A geothermal field, which contains springs across the pH scale (i.e. Rotorua), was closely associated with the highly abundant and prevalent *Acidithiobacillus* and *Venenivibrio*. On the other hand, a predominantly acidic geothermal system (i.e. Te Kopia), produced the only positive associations with “*Methylacidiphilum”* (Verrucomicrobia), *Acidimicrobium* (Actinobacteria), *Terrimonas* (Bacteroidetes), and *Halothiobacillus* (Proteobacteria). These relationships are likely describing sub-community requirements that are otherwise not captured by conventional spatial-statistical analysis, therefore providing insight into previously unrecognised microbe–niche interactions.

### Distance-decay patterns differ at local and regional scales

Environmental selection, ecological drift, diversification, and dispersal limitation all contribute to distance-decay patterns^4^. While several studies have shown microbial dispersal limitations and distance-decay patterns exist in diverse geothermal and non-geothermal environments (e.g. hotsprings^21^, freshwater streams^1^, salt marshes), the point of inflection between dispersal limitation and selection, at regional and local geographic scales, remains under-studied. We identified a positive distance-decay trend with increasing geographic distance between 925 geothermal spring communities across the TVZ region (linear regression: *m* = 0.031, *P* < 0.001; Fig. 4). This finding strongly suggests that dispersal limitation exists between individual geothermal fields. Increasing the resolution to within individual fields, distance-decay patterns are negligible compared to the regional scale (Supplementary Table 6). Interestingly, the greatest pairwise difference (*y* = 1) between Bray–Curtis dissimilarities was also observed in springs classified as geographically-adjacent (< 1.4 m). In the 293 geothermal spring pairs separated by < 1.4 m, temperature had a greater correlation with beta diversity than pH (Spearman’s coefficient: *ρ* = 0.44 and 0.30, respectively, *P* < 0.001). This result illustrates the stark spatial heterogeneity and selective processes that can exist within individual geothermal fields. Congruently, each OTU was detected in an average of only 13 springs (Supplementary Fig. 4). We propose that physical dispersal within geothermal fields is therefore not limiting, but the physicochemical diversity of geothermal springs acts as a barrier to the colonisation of immigrating taxa. However, even between some neighbouring springs with similar (95% CI) geochemical signatures, we did note some dissimilar communities were observed (for example, Waimangu geothermal field; Fig. 3 position E). These differing observations can be explained by either one of three ways: Firstly, the defining parameter driving community structure was not one of the 46 physicochemical variables measured in this study (e.g. dissolved organic carbon or hydrogen); secondly, through the process of dispersal, the differential viability of some extremophilic taxa restricts gene flow and contributes to population genetic drift within geothermal fields^57^. We often consider “extremophilic” microorganisms living in these geothermal environments as the epitome of hardy and robust. In doing so, we overlook that their proximal surroundings (i.e. immediately outside the host spring) may not be conducive to growth and survival^58^ and therefore the divergence of populations in neighbouring, chemically-homogenous spring ecosystems is plausible. Thirdly, aeolian immigration^20^ from the non-geothermal surrounding environment could alter the perceived composition of a community, even when immigrants are not competing to survive. Future work could be undertaken to understand individual population responses to community-wide selective pressures and the temporal nature of ecosystem functioning.

## Conclusion

This study presents data on both niche and neutral drivers of microbial biogeography in 925 geothermal springs at a near-national scale. Our comprehensive data set, with sufficient sampling density and standardised methodology, is the first of its kind to enable a robust spatio-chemical statistical analysis of microbial communities at the regional level across broad physicochemical gradients. Unequivocally, pH drives diversity and community complexity structures within geothermal springs. This effect, however, was only significant at temperatures < 70 °C. We also identified specific taxa associations and finally demonstrated that geochemical signatures can be indicative of community composition. Although a distance-decay pattern across the entire geographic region indicated dispersal limitation, the finding that 293 adjacent community pairs exhibited up to 100% dissimilarity suggests niche selection drives microbial community composition at a localised scale (e.g. within geothermal fields).

This research provides a comprehensive dataset that should be used as a foundation for future studies (e.g. diversification and drift elucidation on targeted spring taxa). It complements the recently published Earth Microbiome Project^44^ by expanding our knowledge of the biogeographical constraints on aquatic ecosystems using standardised quantification of broad physicochemical spectrums. There is also potential to use the two studies to compare geothermal ecosystems on a global scale. Finally, our research provides a springboard to assess the cultural, recreational, and resource development value of the microbial component of geothermal springs, both in New Zealand and globally. Many of the features included in this study occur on culturally-important and protected land for Māori, therefore this or follow-on future projects may provide an avenue for exploration of indigenous knowledge, while assisting in conservation efforts and/or development.

## Methods

### Field sampling and processing

Between July 2013 and April 2015, 1019 aqueous samples were collected from 974 geothermal features within 18 geothermal fields in the TVZ. A 3 L water column sample was taken (to 1 m depth where possible) from each geothermal spring, lake, stream, or the catchment pool of geysers for microbial and chemical analyses. Samples were collected either at the centre of the feature or at ~3 m from the edge to target well-mixed and/or more representative samples, depending on safety and size of the spring. Comprehensive physical and chemical measurements, and field observational metadata were recorded contemporaneously with a custom-built application and automatically uploaded to a database (Supplementary Table 7). All samples were filtered within 2 h of collection and stored accordingly. Total DNA was extracted using a modified CTAB method^59^ with the PowerMag Microbial DNA Isolation Kit using SwiftMag technology (MoBio Laboratories, Carlsbad, CA, USA). The V4 region of the 16S rRNA gene was amplified in triplicate using universal Earth Microbiome Project^44^ primers F515 (5′-GTGCCAGCMGCCGCGGTAA-3′) and R806 (5′-GGACTACVSGGGTATCTAAT-3′), details of which can be found in Supplementary Methods. SPRIselect (Beckman Coulter, Brea, CA, USA) was used to purify DNA following amplification. Amplicon sequencing was performed using the Ion PGM System for Next-Generation Sequencing with the Ion 318v2 Chip and Ion PGM Sequencing 400 Kits (ThermoFisher Scientific, Waltham, MA, USA).

Forty-six separate physicochemical parameters were determined for each geothermal spring sample collected. Inductively coupled plasma–mass spectrometry (ICP-MS) was used to determine the concentrations of aqueous metals and non-metals (31 species; a full list is provided in Supplementary Table 7), and various UV–vis spectrometry methods were used to determine aqueous nitrogen species (${\mathrm{NH}}_4^ +$, ${\mathrm{NO}}_3^ -$, ${\mathrm{NO}}_2^ -$), Fe^2+^, and ${\mathrm{PO}}_4^{3 - }$, with H_2_S, ${\mathrm{HCO}}_3^ -$, and Cl^−^ determined via titration, and SO_4_^2–^ concentration measured via ion chromatography (IC). COND, dO, ORP, pH, and TURB were determined using a Hanna Instruments (Woonsocket, RI, USA) multiparameter fieldmeter at room temperature. Spring temperature (TEMP) was measured in situ immediately after sampling, using a Fluke 51-II thermocouple (Fluke, Everett, WA, USA).

Expanded details on sampling procedures, sample processing, and protocols for DNA extraction, DNA amplification, and chemical analyses can be found in Supplementary Methods and Supplementary Table 7.

### DNA sequence processing

DNA sequences were processed through a custom pipeline utilising components of UPARSE^60^ and QIIME^62^. An initial screening step was performed in mothur^63^ to remove abnormally short (< 275 bp) and long (> 345 bp) sequences. Sequences with long homopolymers (> 6) were also removed. A total of 47,103,077 reads were quality filtered using USEARCH v7^61^ with a maximum expected error of 1% (fastq_maxee = 2.5) and truncated from the forward primer to 250 bp. Retained sequences (85.4% of initial reads) were dereplicated and non-unique sequences removed. Next, reads were clustered to 97% similarity and chimera checked using the cluster_otus command in USEARCH, and a de novo database was created of representative OTUs. 93.2% of the original pre-filtered sequences (truncated to 250 bp) mapped to these OTUs, and taxonomy was assigned using the Ribosomal Database Project Classifier^64^ (with a minimum confidence score of 0.5) against the SILVA 16S rRNA database (123 release, July 2015)^65^. The final read count was 43,202,089, with a mean of 43,905 reads per sample. Chloroplasts and mitochondrial reads were removed (1.0% and 0.5%, respectively of the final read count) and rarefaction was performed to 9500 reads per sample. As a consequence, 94 samples were then removed from the dataset (this included a set of samples collected temporally), leaving a final number of 925 individual samples (see Supplementary Methods). This QC screen also resulted in the removal of one geothermal field from the study (*n* = 17).

### Statistical analyses

All statistical analyses and visualisation were performed in the R environment^66^ using phyloseq^67^, vegan^68^, and ggplot2^69^ packages. Alpha diversity was calculated using the estimate_richness function in phyloseq. A series of filtering criteria were applied to the 46 geochemical parameters measured in this study to identify metadata that significantly correlated with alpha diversity in these spring communities. First, collinear variables (Pearson correlation coefficient |*r*| > 0.7) were detected^70^. The best-fit linear regression between alpha diversity (using Shannon’s index) and each variable was used to pick a representative from each collinear group. This removed variables associated with the same effect in diversity. Multiple linear regression was then applied to remaining variables, before and after a stepwise Akaike information criterion (AIC) model selection was run^71^. Samples were also binned incrementally by pH (single pH units), temperature (10 °C increments) and geographic ranges (geothermal field) (Supplementary Fig. 1), with non-parametric Kruskal–Wallis (*H*) testing performed to identify any significant differences between groups. Finally, correlation of pH and temperature against Shannon diversity was calculated using Pearson’s coefficient |*r*|.

Bray–Curtis dissimilarity was used for all beta diversity comparisons. For ordination visualisations, a square-root transformation was applied to OTU relative abundances prior to non-metric multidimensional scaling (*k* = 2) using the metaMDS function in the vegan package. ANOSIM (|*R*|) was used to compare beta diversity across the same pH, temperature, and geographic groups (i.e. geothermal fields) used for alpha diversity analyses, followed by pairwise Wilcox testing with Bonferroni correction to highlight significance between individual groups. Linear regression was applied to pairwise geographic distances against spring community dissimilarities to assess the significance of distance-decay patterns. These comparisons were similarly performed on spring communities constrained to each geothermal field. A second series of filtering criteria was applied to geochemical parameters to identify metadata that significantly correlated with beta diversity. Mantel tests were performed between beta diversity and all 46 physicochemical variables using Spearman’s correlation coefficient (*ρ*) with 999 permutations. In decreasing order of correlation, metadata were added to a PERMANOVA analysis using the adonis function in vegan. Metadata significantly correlating with beta diversity (*P* < 0.01) was assessed for collinearity using Pearson’s coefficient |*r*|^70^. In each collinear group (|*r*| > 0.7), the variable with the highest mantel statistic was chosen as the representative. Low variant geochemical variables (SD < 0.25 ppm) were then removed to allow a tractable number of explanatory variables for subsequent modelling. Constrained correspondence analysis (using the cca function in vegan) was then applied to OTUs, geothermal field locations, and the reduced set of metadata. OTUs were first agglomerated to their respective genera (using the tax_glom function in phyloseq) and then low abundant taxa (< 0.7% of total mean taxon abundance) were removed. Typical geochemical signatures within each geothermal field were used to produce ternary diagrams of Cl^−^, ${\mathrm{SO}}_4^{2 - }\,{\mathrm{and}}\,{\mathrm{HCO}}_3^ -$ ratios using the ggtern package^72^.

Finally, to detect significant associations between taxa, geochemistry, and other metadata (i.e. geothermal field observations), a multivariate linear model was applied to determine log enrichment of taxa using edgeR^73^. To simplify the display of taxonomy in this model, we first agglomerated all OTUs to their respective genera or closest assigned taxonomy group (using the tax_glom function in phyloseq), and then only used taxa present in at least 5% of samples and > 0.1% average relative abundance. Log fold enrichments of taxa were transformed into *Z*-scores and retained if absolute values were > 1.96. Results were visualised using ggtree^74^. A phylogenetic tree was generated in QIIME by confirming alignment of representative OTU sequences using PyNAST^75^, filtering the alignment to remove positions which were gaps in every sequence and then building an approximately maximum-likelihood tree using FastTree^76^ with a midpoint root.

### Code availability

All code used for statistics and figures is available through GitLab (https://gitlab.com/morganlab/collaboration-1000Springs/1000Springs).

### Data availability

Raw sequences have been deposited into the European Nucleotide Archive (ENA) under study accession number PRJEB24353. A queryable website for the 1000 Springs Project is available at the URL: http://1000springs.org.nz. Other relevant data supporting the findings of the study are available in this article and its Supplementary Information files, or from the corresponding authors upon request.

## Electronic supplementary material

Supplementary Information
