# Supplementary material for: Microbial biogeography of 925 geothermal springs in New Zealand
Source: Nat Commun. 2018 Jul 23;9:2876. doi: 10.1038/s41467-018-05020-y (PMC6056493; doi:10.1038/s41467-018-05020-y)
Supplement: Supplementary file 1 — Supplementary Information [file 41467_2018_5020_MOESM1_ESM.pdf]

# Microbial biogeography of 925 geothermal springs in New Zealand

Jean F. Power[1,2], Carlo R. Carere[1,3], Charles K. Lee[2], Georgia L.J. Wakerley[2], David W. Evans[1], Mathew Button[4], Duncan White[5], Melissa D. Climo[5,6], Annika M. Hinze[4], Xochitl C. Morgan[7], Ian R. McDonald[2], S. Craig Cary[2*] and Matthew B. Stott[1,6*]

[1]Geomicrobiology Research Group, Department of Geothermal Sciences, GNS Science, Taupō 3384, New Zealand
[2]Thermophile Research Unit, School of Science, University of Waikato, Hamilton 3240, New Zealand
[3]Department of Chemical and Process Engineering, University of Canterbury, Christchurch 8140, New Zealand
[4]Department of Computer Science, University of Waikato, Hamilton 3240, New Zealand
[5]Wairakei Research Centre, GNS Science, Taupō 3384, New Zealand
[6]School of Biological Sciences, University of Canterbury, Christchurch 8140, New Zealand
[7]Department of Microbiology and Immunology, University of Otago 9054, Dunedin, New Zealand

***Corresponding authors:** Dr Matthew Stott (matthew.stott@canterbury.ac.nz; +64 (0)3 369 2511) and Prof. Craig Cary (caryc@waikato.ac.nz; +64 (0)7 838 4593)

## SUPPLEMENTARY METHODOLOGY

### Field sampling

Three litres of spring water were collected for each sample taken. A custom-made stainless-steel device was used to capture the water column of each feature to a depth of 1 m where possible, either at the centre of the spring or at ~3 m from the

edge for large features to target well-mixed and/or more representative samples, depending on safety and size of the spring (detailed sketches were drawn of each sampling location to facilitate replication). If the feature was on a slope or the safest sampling position was too far from the spring for the water sampler to operate correctly, a 500 mL PP Nalgene bottle (ThermoFisher Scientific, Waltham, MA, USA) was used to collect the water from the same part of the water column. Either this vessel or the water sampler were then used to aseptically fill a 2000 mL PP Nalgene bottle (ThermoFisher Scientific, Waltham, MA, USA) with spring water for subsequent filtering and DNA extraction. In addition, a 330 mL rubber-sealed glass bottle was collected for geochemical analyses and the 500 mL PP bottle was used to retain water for geophysical parameters. All vessels that contained a microbiological sample were subjected to the same stringent washing procedures, namely with detergent (Extran MA03, EMD Millipore, Billerica, MA, USA) and bleach (20 % v/v bleach:distilled water solution using 5 % sodium hypochlorite), followed by a final autoclave step (122 °C for 20 mins). All metadata were recorded on a custom-made application suitable for Android tablets. Metadata recorded *in situ* at the time of sampling included: sample number, sample date, feature name, feature type, location name, geothermal field, district, latitude and longitude coordinates, detailed description, ebullition, size, colour, spring temperature (same location as sample), and photographs/diagrams of the site. Spring temperature (TEMP) was measured *in situ* immediately after sampling. Parameters measured within two hours of sampling were pH, oxidation-reduction potential (ORP), conductivity (COND), dissolved oxygen (dO), turbidity (TURB), ferrous iron ($Fe^{2+}$) concentration and filtered volume (more details are given in Sample Processing). Entries were digitally linked to the corresponding sample ID and automatically uploaded to an Amazon Relational Database Service (RDS) and E3 Bucket with structured query language (SQL). These results are visible on an Amazon EC2 Web Server, accessed through http://1000springs.org.nz/.

**Sample processing**

Within 2 hours of sampling, the contents of the 2000 mL Nalgene bottle were filtered through a Sterivex-GP 0.22 µm PES column filter (EMD Millipore, Billerica, MA, USA), using the Masterflex E/S Portable Sampler with a peristaltic L/S pump head and platinum-cured silicone L/S tubing (Cole-Parmer, Vernon Hills, IL, USA). All

tubing was bleached and rinsed (first with reverse-osmosis water followed by approximately 150 mL sample water) between samples. Each sample was filtered until all 2000 mL water was pushed through or the filter membrane became clogged. The filters were immediately cooled to 4 °C and then stored at -20 °C until DNA extraction. Filtrate from the column filter was used to fill three 50 mL tubes and two 15 mL tubes with spring water for varying geochemical analyses (Supplementary Table 7). 80 µL of this filtrate was also added to 4 mL ferrous iron reagent (which includes 0.63 mM 2,2-bipyridyl, 0.80 M ammonium acetate and 3.7 % v/v glacial acetic acid)[1] for colorimetric spectroscopy. A multiparameter field meter (Hanna Instruments, Woonsocket, RI, USA) was used to measure pH, oxidation-reduction potential (ORP), conductivity (COND), turbidity (TURB), dissolved oxygen (dO) and sample temperature from the air-tight 500 mL vessel (after samples had cooled to below room temperature). Where pH measured less than 1, a benchtop pH meter (Hanna Instruments, Woonsocket, RI, USA) calibrated to pH 0 was used. All geochemical sample vessels were then stored at either 4 °C or -20 °C until analyses were performed. Aqueous metals and non-metals measured by ICP-MS were Ag, Al, As, B, Ba, Br, Ca, Cd, Co, Cr, Cs, Cu, Fe, Hg, K, Li, Mg, Mn, Mo, Na, Ni, Pb, Rb, S, Se, Si, Sr, Tl, U, V and Zn. Chemical analyses were performed at the Geomicrobiology Research Group (GRG) and the New Zealand Geothermal Analytical Laboratory (NZGAL), both at GNS Science in Wairakei, New Zealand and at the School of Science, University of Waikato, Hamilton, New Zealand. All samples and analyses are summarised in Supplementary Table 7.

**DNA extraction**

DNA extraction, amplification and sequencing were performed at the Thermophile Research Unit and DNA Sequencing Facility (University of Waikato, Hamilton, New Zealand) from a modified cetyl trimethylammonium bromide (CTAB) method[2]. Column filters were thawed, 500 µL of a 0.8 % w/v skim milk powder solution (local consumer brand, freshly prepared for each batch of extractions and treated with UV light for 20 min) added and mixed at 150 RPM on a Ratek orbital mixer at 65 °C for 15 mins. A buffer containing CTAB was used in the extraction lysis buffer which consisted of 2 % (v/v) cetyl trimethylammonium bromide, 1 % (v/v) polyvinyl pyrrolidone, 100 mM Tris-HCl, 1.4 M NaCl and 20 mM EDTA. The final extraction

buffer contained 400 µL CTAB buffer, 200 µL PBS (100 mM) and 100 µL SDS (10 %). This was added to the filters which were then mixed at 150 RPM at 65 °C for 45 mins. The extraction buffer was pushed through the filter and collected. A further 0.7 mL CTAB buffer was added to the filters and mixed at 150 RPM for 15 mins. This filtrate was added to a separate tube, resulting in two extraction duplicates. Chloroform:isoamyl alcohol (24:1), in equal volumes (1:1) to the filtrate, was added to each duplicate and vortexed. These were centrifuged at 10,000 RCF for 12 mins at 4 °C. The aqueous top layers were transferred to new tubes, and 300 µL of chloroform:isoamyl (24:1) added. Again, these were vortexed and centrifuged at 10,000 RCF for 12 mins at 4 °C. The aqueous top layers were removed to fresh tubes. The subsequent steps were modified from the PowerMag Microbial DNA Isolation Kit using SwiftMag technology (MoBio Laboratories, Carlsbad, CA, USA). Equal volumes of 100 % molecular grade ethanol and SwiftMag beads (22:22 µL) were added to the aqueous phases and placed on a magnetic stand for 2 mins. The supernatants were removed and the beads washed with 1 mL of 100 % ethanol. The beads were then resuspended in 22 µL 1X TE (10 mM Tris-HCL containing 1 mM EDTA, pH 8.0). Using a magnet to retain the beads, resuspended DNA were collected, pooled and quantified using the Qubit dsDNA HS assay (ThermoFisher Scientific, Waltham, MA, USA). DNA was then either stored at 4 °C for subsequent PCR that day or at -20 °C for extended periods of time.

**DNA amplification**

PCR reactions were done in triplicate and each final concentration contained: 0.2 µM of forward and reverse primers, 0.016 µg/µL BSA, 0.24 mM of each dNTP, 1.2 X PCR buffer, 6 mM $MgCl_2$, 0.6 U TAQ polymerase and 0.5 ng of DNA to a final volume of 25 µL. Prior to the addition of primers, TAQ polymerase and DNA, the PCR master mix was treated with ethidium monoazide bromide (1 mg/mL stock) to remove exogenous DNA in the PCR reagents[3]. The amount of ethidium monoazide bromide added varied for each batch of the reagent made. This was determined by serial dilution and the resultant highest concentration that did not inhibit PCR (a 1/100 dilution was typical). The master mix was then incubated on ice for 1 min in darkness, followed by 1 min photoactivation using a halogen lamp. All PCR reagents were supplied by Life Technologies (ThermoFisher Scientific, Waltham, MA, USA), except for ethidium monazide bromide (Mediray, Auckland, New Zealand). The

following PCR thermocycling parameters were performed: initial 3 min at 97 °C denaturation, 30 cycles of 45 sec at 94 °C, 60 sec at 50 °C and 90 sec at 72 °C, followed by a final 10 min incubation at 72 °C.

The triplicate amplicons were pooled and purified using SPRIselect (Beckman Coulter, Brea, CA, USA) as per the manufacturer's instructions (recommended ratio of 0.8X SPRI to amplicon volume). Quality and final concentration of the libraries were verified and adjusted to 12 pM via HS Qubit 2.0 (ThermoFisher Scientific, Waltham, MA, USA) and 9100 BioAnalyser (Agilent Technologies, Santa Clara, CA, USA). Amplicon sequencing was then performed using the Ion PGM System for Next-Generation Sequencing (ThermoFisher Scientific, Waltham, MA, USA) with the Ion 318 Chip Kit v2 and 400-base read length chemistry.

**Sample filtering for spatial analyses**

A total of 1,019 samples were taken from 18 different geothermal fields across the entire TVZ for this study from July 2013 to April 2015 (Fig. 1). Twenty-eight of these produced insufficient DNA yields for sequencing. Twenty-two failed to generate adequate sequence reads for downstream processing ($<$ 9,500 reads). Twenty-one geothermal springs were also sampled over time for future investigation of temporal variation ($n = 66$). Forty-four temporal repeats (excluding one already removed due to low sequence reads) were therefore removed from the dataset, leaving a final 925 individual geothermal springs for spatial statistical analyses. This also removed all springs sampled from the geothermal field Atiamuri, which left a final number of 17 individual fields analysed.

**Geochemical filtering**

To build the constrained correspondence analysis (CCA) model, the 46 physicochemical variables measured physicochemistry had to be reduced to a tractable number. Mantel testing of all variables against Bray-Curtis similarities calculated showed significant correlations (Supplementary Table 3). In order of highest to lowest mantel statistic, the variables were added to a permutational multivariate analysis of variance (the adonis function in the vegan package in R). pH and temperature had the highest contributions to this model (12.4 and 3.9 %

respectively, $P < 0.001$, Supplementary Table 4). Manganese, caesium, cadmium, selenium, cobalt, iron, zinc, barium, chromium, calcium and nickel had a $P$-value greater than 0.01 and were removed. Collinear variables were identified in the remaining set (Pearson's coefficient: $|r| > 0.7$) and group representatives with the highest mantel statistic with beta diversity were chosen. This removed rubidium, potassium, vanadium, mercury, sodium, boron, bromine, sulfur, chloride and ferrous iron. The model was re-run on remaining variables and subsequently, magnesium was removed ($P = 0.062$). Of the 24 variables that remained, those with low variation (standard deviation $< 0.25$ ppm) were removed (Supplementary Table 5) – this included strontium, copper, lead, nitrite, molybdenum, thallium, silver and uranium. The remaining 15 variables were added to the CCA model, with geothermal fields and spring communities (Fig. 3). Two springs also had to be removed from this model due to insufficient chemical analyses ($n$ = 923).

## Supplementary References


1. Wilson, A. D. The micro-determination of ferrous iron in silicate minerals by a volumetric and a colorimetric method. *Analyst* **85,** 823–827 (1960).
2. Archer, S. D. J., McDonald, I. R., Herbold, C. W. & Cary, S. C. Characterisation of bacterioplankton communities in the meltwater ponds of Bratina Island, Victoria Land, Antarctica. *FEMS Microbiol. Ecol.* **89,** 451–464 (2014).
3. Rueckert, A. & Morgan, H. W. Removal of contaminating DNA from polymerase chain reaction using ethidium monoazide. *J. Microbiol. Methods* **68,** 596–600 (2007).

## SUPPLEMENTARY TABLES

**Supplementary Table 1** | Linear regression of physicochemistry individually against alpha diversity using the Shannon diversity index.

| | Slope | Std. Error | t-value | *P*-value | Signif. Codes | $R^2$ |
|---|---|---|---|---|---|---|
| pH | 0.1574 | 0.0117 | 13.480 | < 0.0001 | *** | 0.164 |
| $NO_3^-$ | 0.8205 | 0.0834 | 9.838 | < 0.0001 | *** | 0.095 |
| $SO_4^{2-}$ | -0.0003 | 0.0000 | -6.620 | < 0.0001 | *** | 0.045 |
| TURB | -0.0009 | 0.0001 | -6.382 | < 0.0001 | *** | 0.042 |
| $H_2S$ | -0.0140 | 0.0026 | -5.304 | < 0.0001 | *** | 0.030 |
| Al | -0.0049 | 0.0010 | -5.100 | < 0.0001 | *** | 0.027 |
| dO | 0.0727 | 0.0148 | 4.899 | < 0.0001 | *** | 0.025 |
| S | -0.0006 | 0.0001 | -4.675 | < 0.0001 | *** | 0.023 |
| U | -350.7932 | 76.2842 | -4.599 | < 0.0001 | *** | 0.022 |
| $HCO_3^-$ | 0.0011 | 0.0003 | 4.229 | < 0.0001 | *** | 0.019 |
| Li | 0.0321 | 0.0080 | 4.009 | < 0.0001 | *** | 0.017 |
| Na | 0.0004 | 0.0001 | 3.887 | < 0.0001 | *** | 0.016 |
| As | 0.0627 | 0.0195 | 3.216 | 0.0013 | ** | 0.011 |
| Cs | 0.1131 | 0.0373 | 3.030 | 0.0025 | ** | 0.010 |
| K | 0.0020 | 0.0007 | 2.831 | 0.0047 | ** | 0.009 |
| Br | 0.0355 | 0.0133 | 2.664 | 0.0079 | ** | 0.008 |
| Se | 14.3009 | 5.4569 | 2.621 | 0.0089 | ** | 0.007 |
| ORP | -0.0005 | 0.0002 | -2.596 | 0.0096 | ** | 0.007 |
| $NH_4^+$ | -0.0014 | 0.0005 | -2.594 | 0.0096 | ** | 0.007 |
| Rb | 0.1298 | 0.0534 | 2.429 | 0.0153 | * | 0.006 |
| Fe | -0.0027 | 0.0012 | -2.319 | 0.0206 | * | 0.006 |
| $Fe^{2+}$ | -0.0023 | 0.0010 | -2.317 | 0.0207 | * | 0.006 |
| $NO_2^-$ | 0.9100 | 0.4052 | 2.246 | 0.0250 | * | 0.005 |
| B | 0.0051 | 0.0023 | 2.188 | 0.0289 | * | 0.005 |
| Mn | -0.0555 | 0.0297 | -1.870 | 0.0619 | . | 0.004 |
| V | -0.6407 | 0.3791 | -1.690 | 0.0913 | . | 0.003 |
| Si | -0.0010 | 0.0006 | -1.656 | 0.0980 | . | 0.003 |
| Cd | 36.1309 | 23.0894 | 1.565 | 0.1180 | | 0.003 |
| Co | -9.3257 | 6.3612 | -1.466 | 0.1430 | | 0.002 |
| Cr | -0.2915 | 0.2145 | -1.359 | 0.1740 | | 0.002 |
| TEMP | 0.0018 | 0.0014 | 1.279 | 0.2010 | | 0.002 |
| Ca | -0.0005 | 0.0004 | -1.175 | 0.2400 | | 0.001 |
| Ni | -1.0608 | 0.9284 | -1.143 | 0.2540 | | 0.001 |

| | | | | | |
|---|---|---|---|---|---|
| Mo | 2.7628 | 2.4559 | 1.125 | 0.2610 | 0.001 |
| COND | 0.0000 | 0.0000 | -1.098 | 0.2720 | 0.001 |
| Hg | -2.3407 | 2.1961 | -1.066 | 0.2870 | 0.001 |
| Mg | -0.0009 | 0.0008 | -1.022 | 0.3070 | 0.001 |
| Ba | -0.0648 | 0.0639 | -1.015 | 0.3100 | 0.001 |
| Ag | -43.2878 | 47.1283 | -0.919 | 0.3590 | 0.001 |
| Zn | -0.1021 | 0.1115 | -0.916 | 0.3600 | 0.001 |
| $Cl^-$ | 0.0000 | 0.0000 | -0.700 | 0.4840 | 0.001 |
| Tl | 5.0194 | 7.6863 | 0.653 | 0.5140 | 0.000 |
| $PO_4^{3-}$ | 0.0269 | 0.1135 | 0.237 | 0.8130 | 0.000 |
| Pb | -0.0390 | 0.3551 | -0.110 | 0.9130 | 0.000 |
| Sr | 0.0100 | 0.1433 | 0.070 | 0.9440 | 0.000 |
| Cu | 0.0149 | 0.2460 | 0.060 | 0.9520 | 0.000 |

Signif. codes: 0-0.001 '***'; 0.001-0.01 '**'; 0.01-0.05 '*'; 0.05-0.1 '.'; 0.1-1 ' '

TURB: turbidity, dO: dissolved oxygen, ORP: oxidation-reduction potential, COND: conductivity

**Supplementary Table 2 |** Multiple linear regression model of significant physicochemical parameters against alpha diversity (Shannon Index), after collinear variables were removed and an Akaike information criterion (AIC) was applied. Variables were added to the model in order of highest to lowest best fit from singular linear regression.

| | Estimate | Std. Error | t-value | *P*-value | Signif. codes |
|---|---|---|---|---|---|
| (Intercept) | 2.7116 | 0.1107 | 24.502 | < 0.0001 | *** |
| pH | 0.1568 | 0.0140 | 11.201 | < 0.0001 | *** |
| $NO_3^-$ | 0.7690 | 0.0905 | 8.502 | < 0.0001 | *** |
| TURB | -0.0002 | 0.0001 | -1.768 | 0.0774 | . |
| dO | 0.0498 | 0.0131 | 3.807 | 0.0002 | *** |
| ORP | 0.0004 | 0.0002 | 2.219 | 0.0268 | * |
| $NO_2^-$ | -1.6099 | 0.4104 | -3.923 | 0.0001 | *** |
| Si | -0.0014 | 0.0005 | -2.689 | 0.0073 | ** |
| Cd | 48.7715 | 19.8181 | 2.461 | 0.0140 | * |

Multiple R-squared: 0.273, adjusted R-squared: 0.2666

F-statistic: 42.99 on 8 and 916 degrees of freedom, p-value: < 2.2e-16

Signif. codes: 0-0.001 '***'; 0.001-0.01 '**'; 0.01-0.05 '*'; 0.05-0.1 '.'; 0.1-1 ' '

TURB: turbidity, dO: dissolved oxygen

**Supplementary Table 3 |** Mantel tests using Spearman's correlation (permutations = 999) of Bray-Curtis dissimilarities between all communities sampled and each individual geochemical parameter and geographic distance (km).

| | Rho (ρ) | *P*-value | Signif. codes |
|---|---|---|---|
| pH | 0.544 | < 0.001 | *** |
| $SO_4^{2-}$ | 0.248 | < 0.001 | *** |
| Al | 0.224 | < 0.001 | *** |
| TEMP | 0.193 | < 0.001 | *** |
| $HCO_3^-$ | 0.153 | < 0.001 | *** |
| TURB | 0.145 | < 0.001 | *** |
| S | 0.131 | < 0.001 | *** |
| km | 0.122 | < 0.001 | *** |
| $NH_4^+$ | 0.103 | < 0.001 | *** |
| As | 0.102 | < 0.001 | *** |
| COND | 0.098 | < 0.001 | *** |
| Mo | 0.086 | < 0.001 | *** |
| Fe | 0.082 | < 0.001 | *** |
| Li | 0.082 | < 0.001 | *** |
| Ag | 0.079 | < 0.001 | *** |
| Tl | 0.074 | < 0.001 | *** |
| $NO_2^-$ | 0.073 | < 0.001 | *** |
| V | 0.072 | < 0.001 | *** |
| U | 0.069 | < 0.001 | *** |
| Rb | 0.068 | < 0.001 | *** |
| Na | 0.068 | < 0.001 | *** |
| Cs | 0.064 | < 0.001 | *** |
| Mn | 0.063 | < 0.001 | *** |
| B | 0.059 | < 0.001 | *** |
| K | 0.058 | < 0.001 | *** |
| Mg | 0.057 | < 0.001 | *** |
| ORP | 0.057 | < 0.001 | *** |
| $Cl^-$ | 0.056 | < 0.001 | *** |
| $Fe^{2+}$ | 0.056 | < 0.001 | *** |
| Hg | 0.055 | < 0.001 | *** |
| $NO_3^-$ | 0.055 | < 0.001 | *** |
| Si | 0.046 | < 0.001 | *** |
| Sr | 0.044 | < 0.001 | *** |
| Co | 0.040 | 0.002 | ** |

| | | | |
|---|---|---|---|
| Br | 0.038 | 0.002 | ** |
| Zn | 0.044 | < 0.001 | *** |
| dO | 0.041 | < 0.001 | *** |
| Se | 0.031 | 0.003 | ** |
| Ca | 0.029 | 0.007 | ** |
| Ba | 0.020 | 0.017 | * |
| Cd | 0.020 | 0.068 | . |
| $H_2S$ | 0.014 | 0.142 | |
| Cr | 0.007 | 0.276 | |
| Pb | 0.006 | 0.297 | |
| $PO_4^{3-}$ | 0.005 | 0.344 | |
| Cu | -0.008 | 0.736 | |
| Ni | -0.020 | 0.953 | |

Signif. codes:  0-0.001 '***'; 0.001-0.01 '**'; 0.01-0.05 '*'; 0.05-0.1 '.'; 0.1-1 ' '

TEMP: temperature, TURB: turbidity, km: kilometres, COND: conductivity, dO: dissolved oxygen, ORP: oxidation reduction potential

**Supplementary Table 4 |** Permutational multivariate analysis of variance (using continuous variables only) of beta diversity using Bray-Curtis dissimilarities.

| | Df | SumsOfSqs | MeanSqs | F.Model | $R^2$ | $P$-value | Signif. codes |
|---|---|---|---|---|---|---|---|
| pH | 1 | 44.13 | 44.125 | 154.945 | 0.124 | 0.001 | *** |
| TEMP | 1 | 14.03 | 14.026 | 49.253 | 0.039 | 0.001 | *** |
| ORP | 1 | 5.06 | 5.056 | 17.755 | 0.014 | 0.001 | *** |
| $SO_4^{2-}$ | 1 | 2.88 | 2.883 | 10.123 | 0.008 | 0.001 | *** |
| TURB | 1 | 2.71 | 2.708 | 9.508 | 0.008 | 0.001 | *** |
| As | 1 | 2.51 | 2.512 | 8.820 | 0.007 | 0.001 | *** |
| $NO_3^-$ | 1 | 2.25 | 2.249 | 7.896 | 0.006 | 0.001 | *** |
| $NH_4^+$ | 1 | 1.99 | 1.989 | 6.986 | 0.006 | 0.001 | *** |
| $HCO_3^-$ | 1 | 1.84 | 1.839 | 6.457 | 0.005 | 0.001 | *** |
| Rb | 1 | 1.76 | 1.757 | 6.169 | 0.005 | 0.001 | *** |
| Ag | 1 | 1.39 | 1.389 | 4.877 | 0.004 | 0.001 | *** |
| $NO_2^-$ | 1 | 1.25 | 1.254 | 4.405 | 0.004 | 0.001 | *** |
| K | 1 | 1.22 | 1.223 | 4.294 | 0.003 | 0.001 | *** |
| COND | 1 | 1.14 | 1.143 | 4.014 | 0.003 | 0.001 | *** |
| dO | 1 | 1.10 | 1.096 | 3.848 | 0.003 | 0.001 | *** |
| $H_2S$ | 1 | 1.00 | 1.001 | 3.514 | 0.003 | 0.001 | *** |

| | | | | | | | |
|---|---|---|---|---|---|---|---|
| Tl | 1 | 1.00 | 0.998 | 3.506 | 0.003 | 0.001 | *** |
| V | 1 | 0.96 | 0.959 | 3.369 | 0.003 | 0.001 | *** |
| Mg | 1 | 0.88 | 0.880 | 3.090 | 0.002 | 0.001 | *** |
| Al | 1 | 0.84 | 0.838 | 2.942 | 0.002 | 0.001 | *** |
| Si | 1 | 0.83 | 0.830 | 2.916 | 0.002 | 0.001 | *** |
| Sr | 1 | 0.75 | 0.753 | 2.643 | 0.002 | 0.001 | *** |
| Hg | 1 | 0.72 | 0.718 | 2.522 | 0.002 | 0.001 | *** |
| Mo | 1 | 0.64 | 0.640 | 2.247 | 0.002 | 0.001 | *** |
| Pb | 1 | 0.56 | 0.565 | 1.984 | 0.002 | 0.001 | *** |
| Na | 1 | 1.02 | 1.020 | 3.582 | 0.003 | 0.002 | ** |
| B | 1 | 0.95 | 0.945 | 3.320 | 0.003 | 0.002 | ** |
| Br | 1 | 0.88 | 0.880 | 3.089 | 0.002 | 0.002 | ** |
| Li | 1 | 0.87 | 0.866 | 3.041 | 0.002 | 0.002 | ** |
| $PO_4^{3-}$ | 1 | 0.65 | 0.653 | 2.292 | 0.002 | 0.002 | ** |
| Cu | 1 | 0.61 | 0.612 | 2.149 | 0.002 | 0.002 | ** |
| U | 1 | 0.74 | 0.744 | 2.613 | 0.002 | 0.003 | ** |
| S | 1 | 0.83 | 0.827 | 2.903 | 0.002 | 0.004 | ** |
| $Cl^-$ | 1 | 0.75 | 0.748 | 2.628 | 0.002 | 0.005 | ** |
| $Fe^{2+}$ | 1 | 0.57 | 0.568 | 1.995 | 0.002 | 0.005 | ** |
| Mn | 1 | 0.58 | 0.581 | 2.040 | 0.002 | 0.011 | * |
| Cs | 1 | 0.59 | 0.592 | 2.078 | 0.002 | 0.014 | * |
| Cd | 1 | 0.39 | 0.394 | 1.384 | 0.001 | 0.019 | * |
| Se | 1 | 0.47 | 0.473 | 1.660 | 0.001 | 0.055 | . |
| Co | 1 | 0.38 | 0.379 | 1.329 | 0.001 | 0.142 | |
| Fe | 1 | 0.36 | 0.357 | 1.255 | 0.001 | 0.145 | |
| Zn | 1 | 0.37 | 0.367 | 1.289 | 0.001 | 0.146 | |
| Ba | 1 | 0.37 | 0.369 | 1.296 | 0.001 | 0.172 | |
| Cr | 1 | 0.35 | 0.355 | 1.247 | 0.001 | 0.187 | |
| Ca | 1 | 0.34 | 0.344 | 1.209 | 0.001 | 0.225 | |
| Ni | 1 | 0.23 | 0.227 | 0.797 | 0.001 | 0.758 | |

Signif. codes: 0-0.001 ‘***’; 0.001-0.01 ‘**’; 0.01-0.05 ‘*’; 0.05-0.1 ‘.’; 0.1-1 ‘ ‘

TURB: turbidity, dO: dissolved oxygen, ORP: oxidation-reduction potential, COND: conductivity

**Supplementary Table 5 |** Variance and standard deviation (SD) of significant chemistry (ppm) correlating with beta diversity. Parameters with SD < 0.25 ppm were removed for building the constrained correspondence analysis (CCA) model with a tractable number of variables.

| | **Variance ($\sigma^2$)** | **SD (ppm)** |
|---|---|---|
| $SO_4^{2-}$ | 404969.24 | 636.37 |
| $HCO_3^-$ | 15489.52 | 124.46 |
| $NH_4^+$ | 3417.50 | 58.46 |
| Si | 2945.29 | 54.27 |
| Al | 452.90 | 21.28 |
| $H_2S$ | 141.45 | 11.89 |
| Li | 15.49 | 3.94 |
| As | 2.58 | 1.61 |
| $NO_3^-$ | 0.13 | 0.36 |
| $PO_4^{3+}$ | 0.08 | 0.28 |
| Sr | 0.04 | 0.20 |
| Cu | 0.02 | 0.13 |
| Pb | 0.01 | 0.09 |
| $NO_2^-$ | 0.01 | 0.08 |
| Mo | 0.00 | 0.01 |
| Tl | 0.00 | 0.00 |
| Ag | 0.00 | 0.00 |
| U | 0.00 | 0.00 |

**Supplementary Table 6** | Linear regression of spring community dissimilarity in each geothermal field against geographic distance. Geothermal fields Whangairorohea, Ohaaki and Misc were removed from this analysis due to low spring numbers present ($n < 3$). Fields are ordered north to south.

| | **Slope** | **Std. Error** | **t-value** | **$P$-value** | **Signif. Codes** | **$R^2$** |
|---|---|---|---|---|---|---|
| White Island | 0.0003 | 0.0001 | 2.925 | 0.0042 | ** | 0.077 |
| Taheke | 0.0003 | 0.0003 | 0.995 | 0.3490 | | 0.110 |
| Tikitere | 0.0000 | 0.0000 | 2.570 | 0.0102 | * | 0.002 |
| Rotorua | 0.0000 | 0.0000 | 25.850 | < 0.0001 | *** | 0.010 |
| Waimangu | 0.0000 | 0.0000 | 1.858 | 0.0632 | . | 0.001 |
| Waikite | 0.0001 | 0.0000 | 4.389 | 0.0000 | *** | 0.046 |

| | | | | | | |
|---|---|---|---|---|---|---|
| Waiotapu | 0.0000 | 0.0000 | 10.110 | < 0.0001 | *** | 0.040 |
| Te Kopia | 0.0000 | 0.0001 | -0.117 | 0.9070 | | 0.000 |
| Reporoa | 0.0000 | 0.0000 | 6.541 | 0.0000 | *** | 0.096 |
| Orakei Korako | 0.0001 | 0.0001 | 1.686 | 0.0929 | . | 0.010 |
| Ngatamariki | 0.0001 | 0.0001 | 2.566 | 0.0111 | * | 0.035 |
| Rotokawa | 0.0001 | 0.0000 | 9.565 | < 0.0001 | *** | 0.041 |
| Wairakei-Tauhara | 0.0000 | 0.0000 | 21.200 | < 0.0001 | *** | 0.153 |
| Tokaanu | 0.0005 | 0.0000 | 12.280 | < 0.0001 | *** | 0.185 |

Signif. codes: 0-0.001 ‘***’; 0.001-0.01 ‘**’; 0.01-0.05 ‘*’; 0.05-0.1 ‘.’; 0.1-1 ‘ ‘

**Supplementary Table 7 |** A list of all DNA and physicochemical samples taken from each spring, and the subsequent processing and analyses performed for each individual parameter.

| **Parameter** | **Volume (mL)** | **Processing** | **Storage** | **Analytical Method** |
|---|---|---|---|---|
| Microbial diversity | 2,000 | Filtered | -20 °C | DNA extraction, sequencing |
| Temperature | NA | Raw | NA (*in situ*) | Digital thermocouple |
| *Physical properties | 500 | Raw | RT | Multiparameter field meter |
| $Fe^{2+}$ | 0.08 | Filtered | RT | Colorimetric spectroscopy |
| $H_2S$ | 330 | Raw | 4 °C | Iodometric titration |
| $HCO_3^-$ | 330 | Raw | 4 °C | $HCO_3$ titration |
| $Cl^-$ | 330 | Raw | 4 °C | Potentiometric titration/IC |
| $SO_4^{2-}$ | 50 | Filtered | 4 °C | IC |
| $NH_4^+$, $PO_4^{3-}$, $NO_2^-$, $NO_3^-$ | 50 | Filtered | -20 °C | FIA |
| Back up sample | 50 | Filtered | 4 °C | NA |
| **Elemental analysis | 15 | Filtered, acidified | 4 °C | ICP-MS |

**NA:** not applicable, **RT:** room temperature, **IC:** ion chromatography, **FIA:** flow injection analysis, **ICP-MS:** inductively coupled plasma-mass spectroscopy.

*Physical properties measured by the field meter were pH, oxidation-reduction potential (ORP), conductivity (COND), turbidity (TURB) and dissolved oxygen (dO)

**Elements measured using ICP-MS were Al, Ag, As, B, Ba, Br, Ca, Cd, Co, Cr, Cu, Cs, Fe, Hg, K, Li, Mg, Mo, Mn, Na, Ni, Pb, Rb, S, Se, Si, Sr, Tl, U, V and Zn.

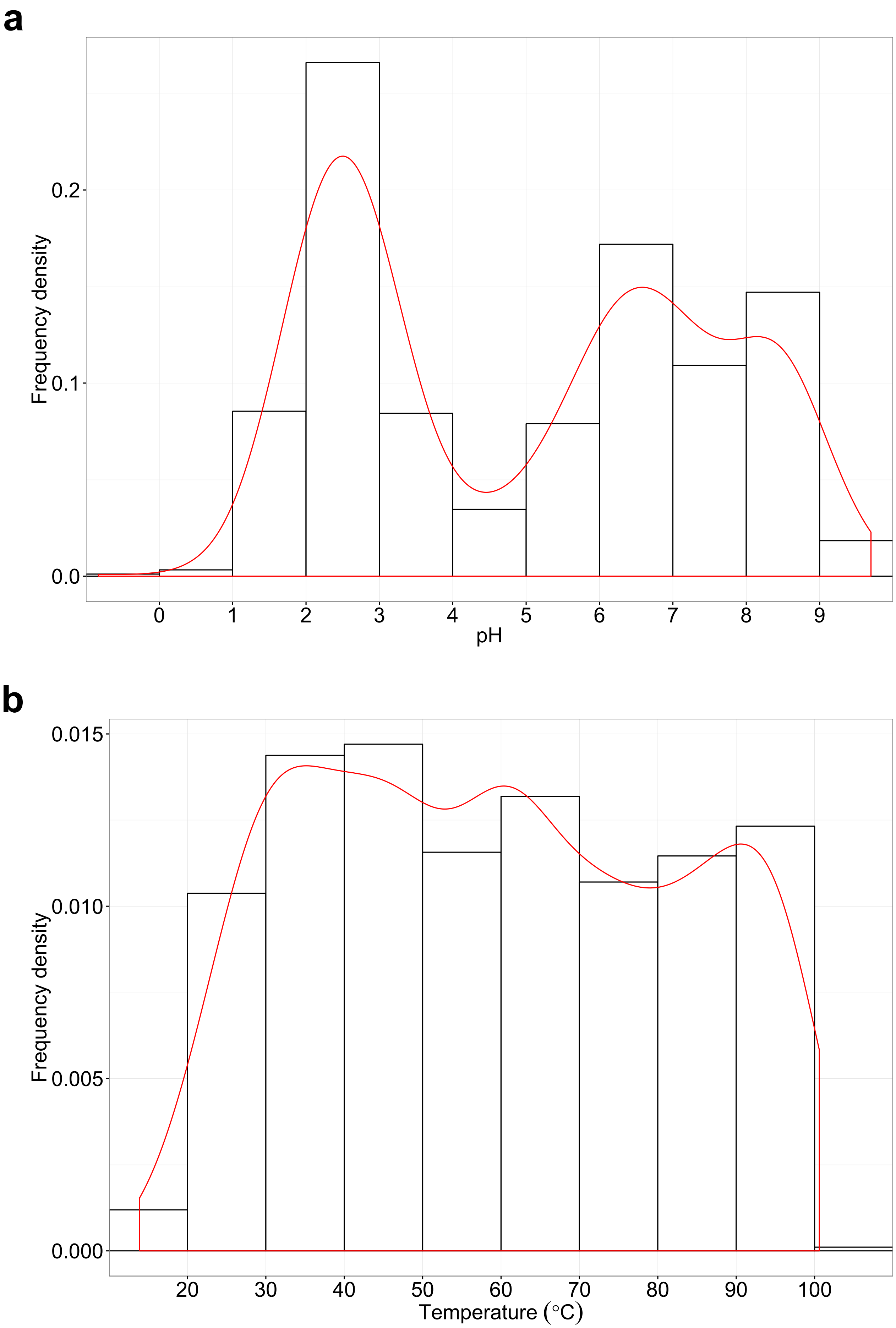


**Supplementary Fig. 1 | pH (a) and temperature (b) frequencies from all spring communities sampled.** Red trendlines are a function of frequency density ($n$ = 925).

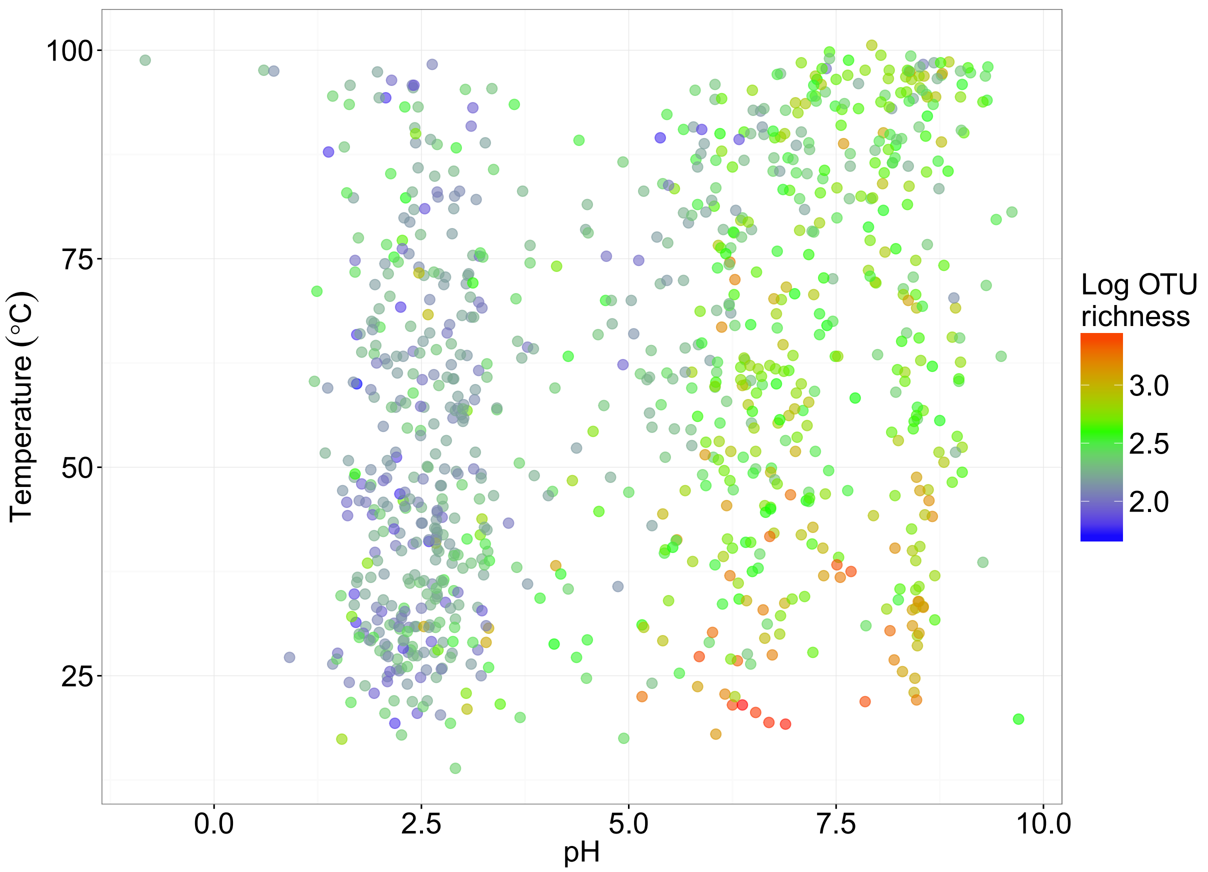


**Supplementary Fig. 2 | pH, temperature and alpha diversity scales.** A scatter plot of pH and temperature gradients for all springs sampled ($n$ = 925). The number of OTUs or richness is shown in colour (range: 49 – 2997, mean: 386, median: 247).

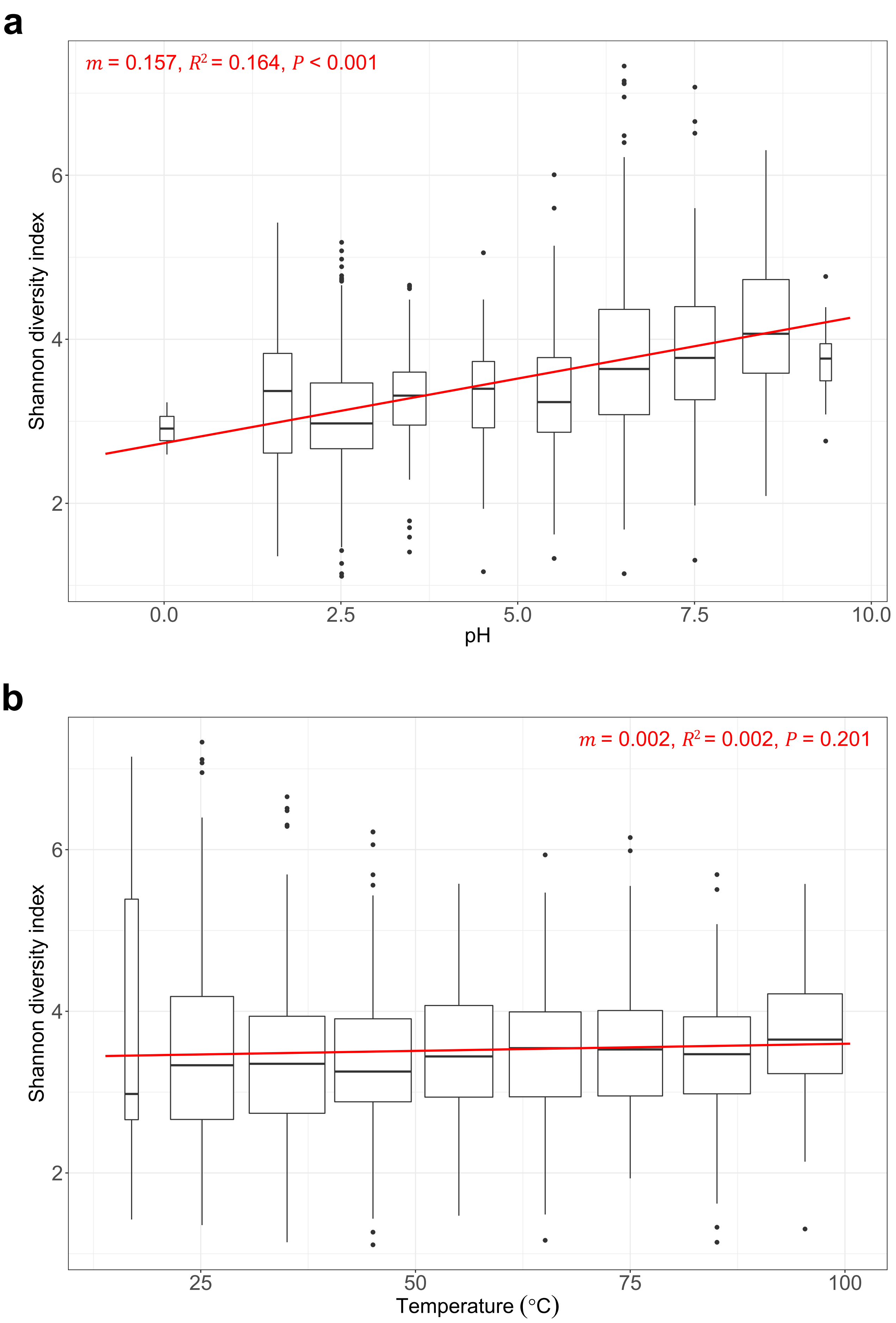


**Supplementary Fig. 3 | Alpha diversity against pH (a) and temperature (b).** Linear regression of Shannon index against each variable is shown in red ($n$ = 925).

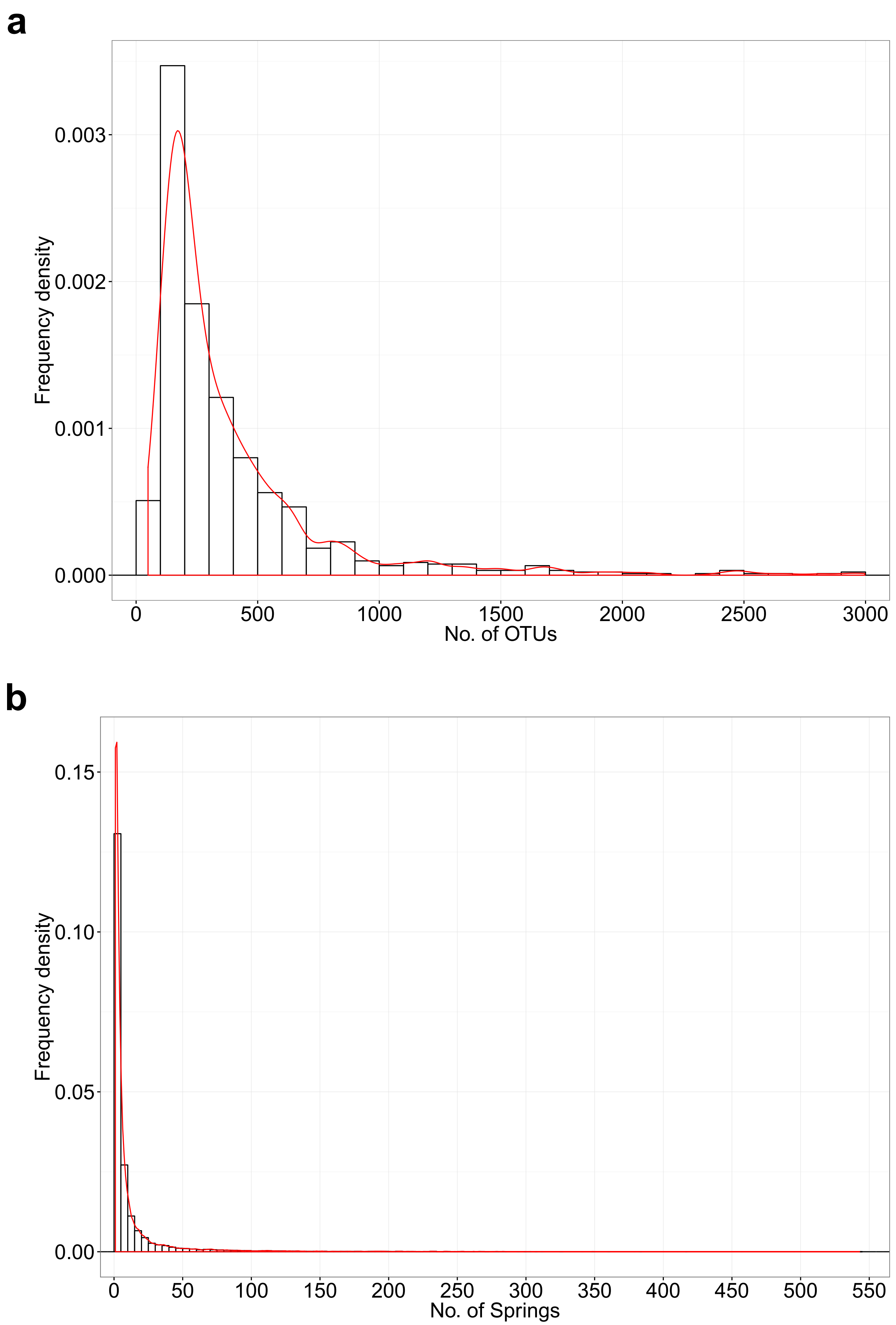


**Supplementary Fig. 4 | Quantity and distribution of OTUs across springs.**
(a) Histogram shows the number of OTUs found in each spring ($n$ = 28,381, range: 49-2997, mean: 386, median: 247). (b) The distribution of each OTU across all springs ($n$ = 925, range: 1-547, mean: 13, median: 3).

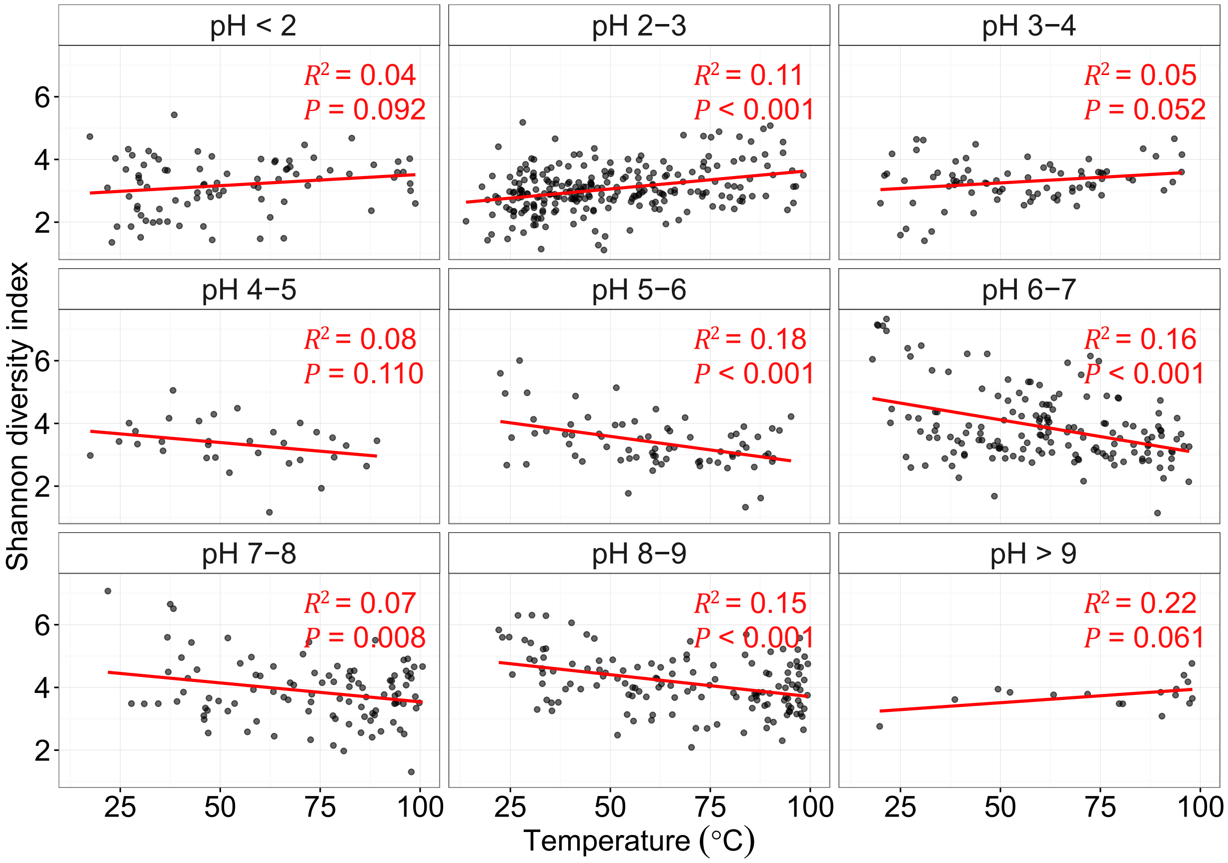


**Supplementary Fig. 5 | The relationship between alpha diversity and temperature.** Samples ($n$ = 925) are split into pH increments. Linear regression of each increment against Shannon diversity index is shown in red.

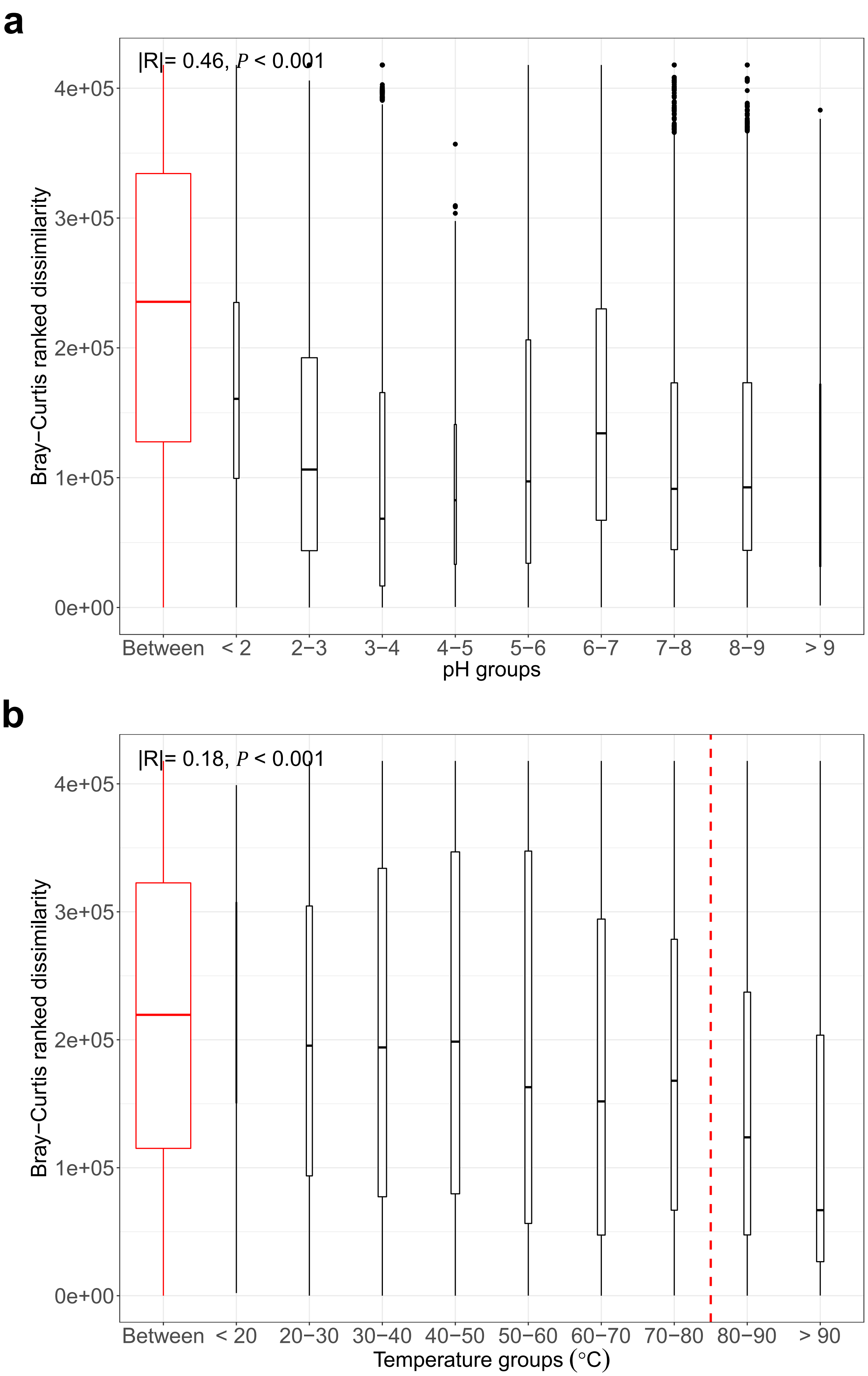


**Supplementary Fig. 6 | Analysis of similarities (ANOSIM: |R|) of pH and temperature binned samples ($n$ = 925) against beta diversity.** The variation between pH (a) and temperature (b) groups is shown in red, with the variation within individual groups in black. The red dashed line in (b) refers to the greatest significant difference between individual temperature groups (Wilcoxon test: $P < 2 \times 10^{-16}$; > 80 °C).

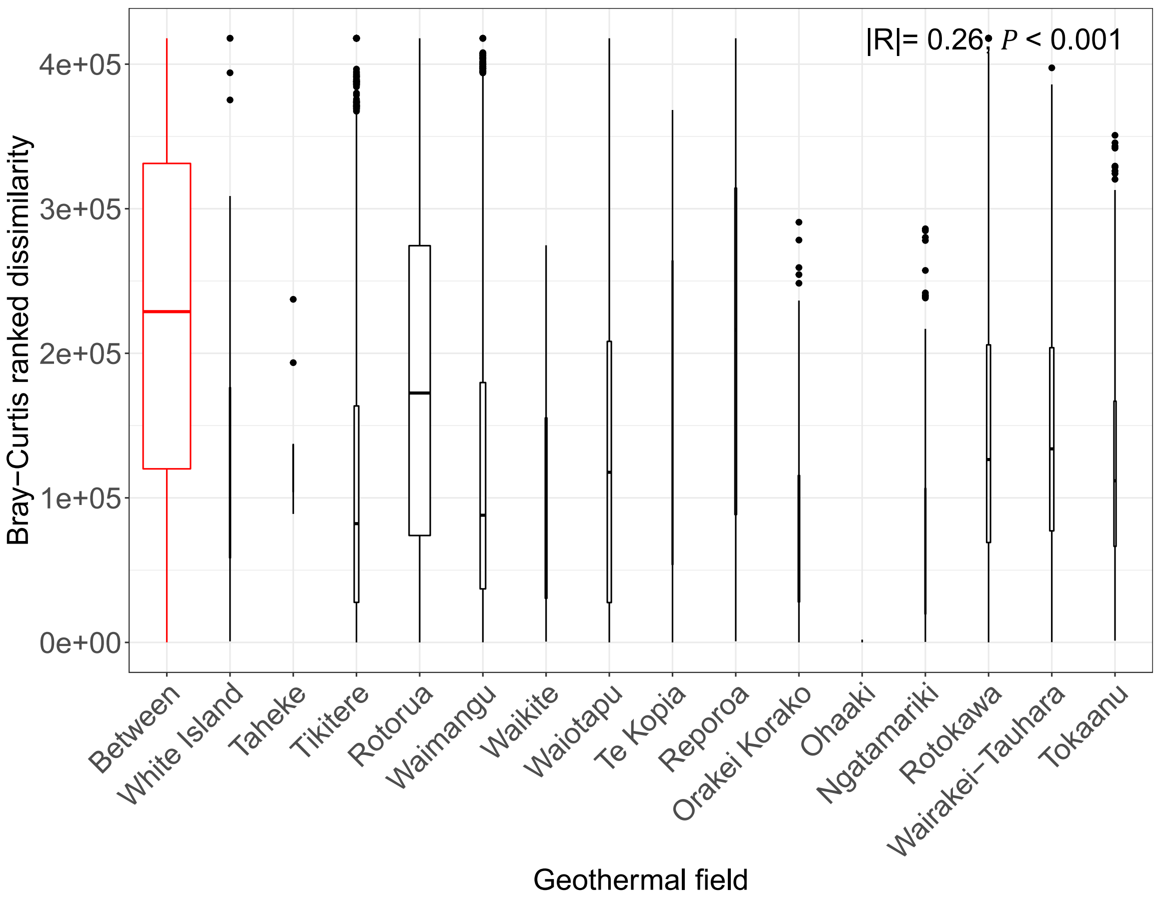


**Supplementary Fig. 7 | Analysis of similarities (ANOSIM: |R|) between geothermal fields and beta diversity.** The overall variation between fields is plotted in red, with the variation within each individual field in black. The width of each bar indicates the number of springs ($n$ = 925) per site. Two geothermal fields (Whangairorohea and Misc) had only one spring sampled and therefore are not shown in this analysis.

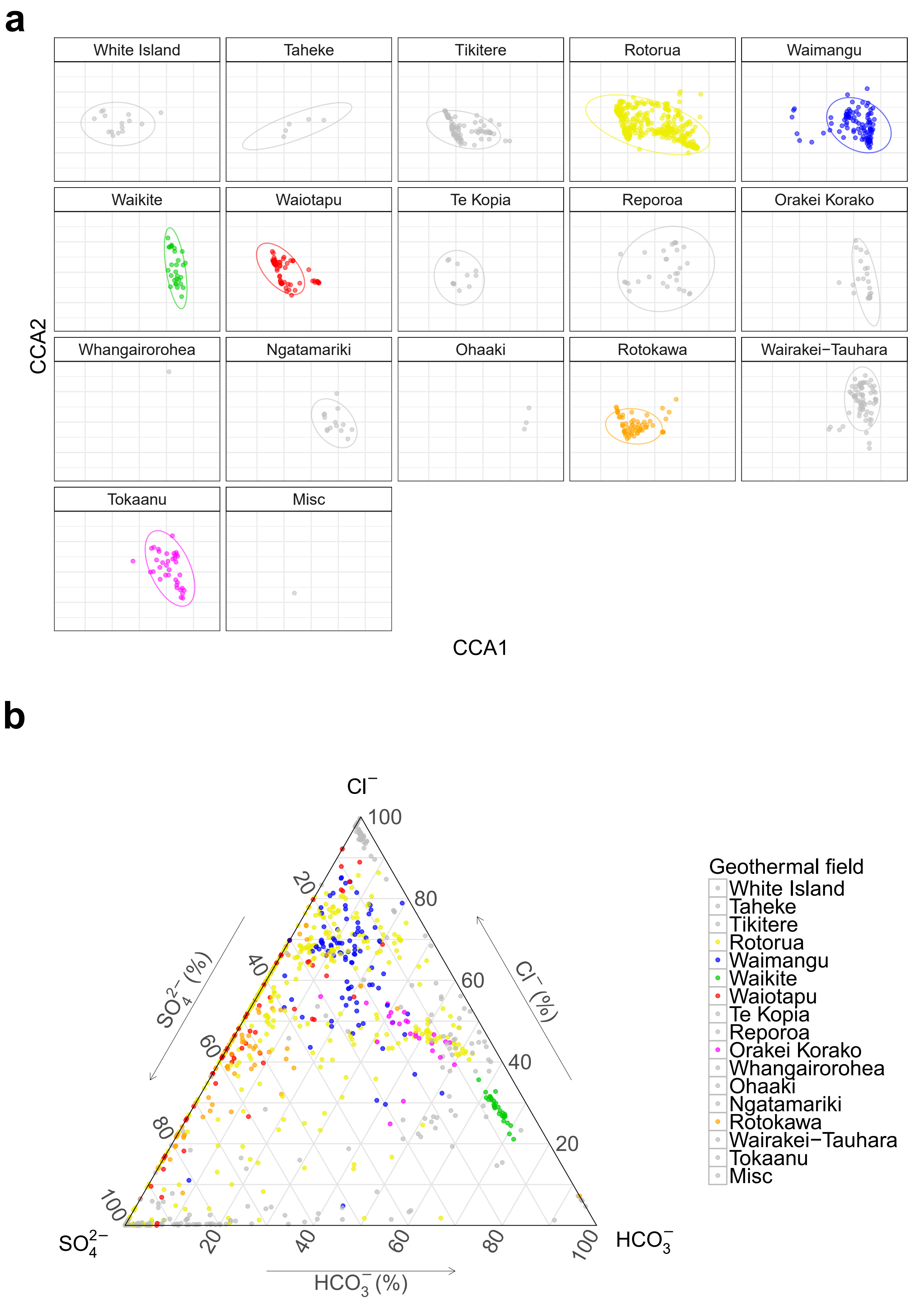


**Supplementary Fig. 8 | Constrained correspondence analysis (CCA) of geothermal fields and geochemical signatures.** (a) CCA ordination for each geothermal field – full model is shown in Fig. 3 ($n$ = 923). Ellipses are plotted as 95 % confidence intervals. (b) Ternary diagram of geochemical signatures for springs sampled ($n$ = 923). Points are colour-coded to geothermal fields shown individually in Fig. 3.

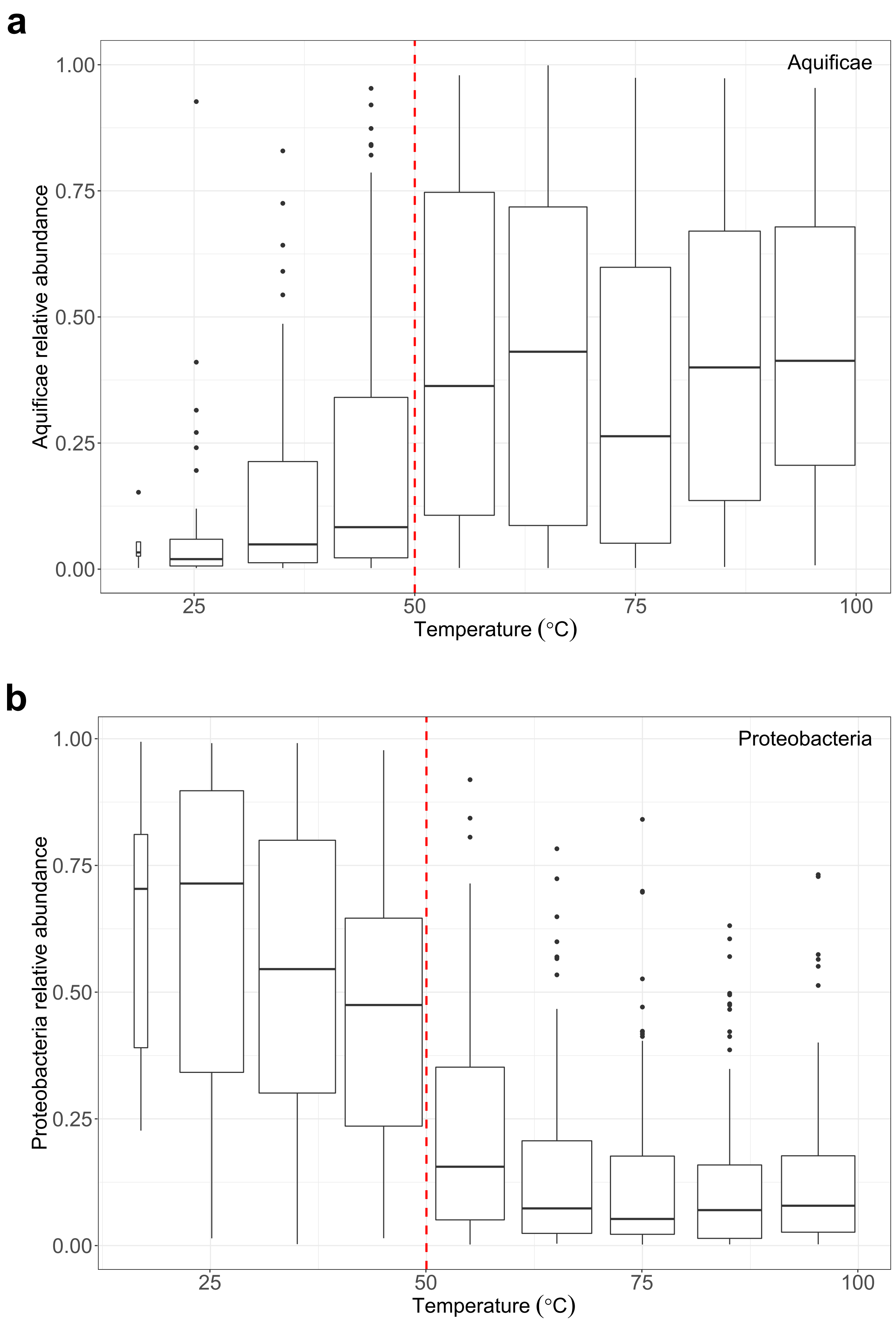


**Supplementary Fig. 9 | Relative abundance of Aquificae (a) and Proteobacteria (b) in each spring against temperature.** The boxplots are binned by 10 °C increments. The red dotted line marks a substantial change in abundance levels at 50 °C ($n$ = 925 for both plots).
